# Selective use of primate CD4 receptors by HIV-1

**DOI:** 10.1371/journal.pbio.3000304

**Published:** 2019-06-10

**Authors:** Cody J. Warren, Nicholas R. Meyerson, Obaiah Dirasantha, Emily R. Feldman, Gregory K. Wilkerson, Sara L. Sawyer

**Affiliations:** 1 BioFrontiers Institute, Department of Molecular, Cellular, and Developmental Biology, University of Colorado, Boulder, Colorado, United States of America; 2 Department of Comparative Medicine, Michale E. Keeling Center for Comparative Medicine and Research, The University of Texas MD Anderson Cancer Center, Bastrop, Texas, United States of America; Northwestern University Feinberg School of Medicine, UNITED STATES

## Abstract

Individuals chronically infected with HIV-1 harbor complex viral populations within their bloodstreams. Recently, it has come to light that when these people infect others, the new infection is typically established by only one or a small number of virions from within this complex viral swarm. An important goal is to characterize the biological properties of HIV-1 virions that seed and exist early in new human infections because these are potentially the only viruses against which a prophylactic HIV-1 vaccine would need to elicit protection. This includes understanding how the Envelope (Env) protein of these virions interacts with the T-cell receptor CD4, which supports attachment and entry of HIV-1 into target cells. We examined early HIV-1 isolates for their ability to infect cells via the CD4 receptor of 15 different primate species. Primates were the original source of HIV-1 and now serve as valuable animal models for studying HIV-1. We find that most primary isolates of HIV-1 from the blood, including early isolates, are highly selective and enter cells through some primate CD4 receptor orthologs but not others. This phenotype is remarkably consistent, regardless of route of transmission, viral subtype, or time of isolation post infection. We show that the weak CD4 binding affinity of blood-derived HIV-1 isolates is what makes them sensitive to the small sequence differences in CD4 from one primate species to the next. To substantiate this, we engineered an early HIV-1 Env to have high, medium, or low binding affinity to CD4, and we show that it loses the ability to enter cells via the CD4 receptor of many primate species as the binding affinity gets weaker. Based on the phenotype of selective use of primate CD4, we find that weak CD4 binding appears to be a nearly universal property of HIV-1 circulating in the bloodstream. Therefore, weak binding to CD4 must be a selected and important property in the biology of HIV-1 in the body. We identify six primate species that encode CD4 receptors that fully support the entry of early HIV-1 isolates despite their low binding affinity for CD4. These findings will help inform long-standing efforts to model HIV-1 transmission and early disease in primates.

## Abstract 2

The current animal model for HIV, the macaque, encodes a CD4 receptor that is non-permissive for HIV entry. This paper reveals that six primate species encode CD4 receptors compatible with HIV infection, potentially making them powerful tools for the study of HIV biology. Furthermore, weak CD4 binding is a nearly constant, and apparently selected, property of HIV circulating in the human bloodstream.

## Introduction

Individuals chronically infected with HIV-1 harbor complex viral populations within their bloodstreams. Recently, it has come to light that when these people infect others, the new infection is typically established by only one or a small number of virions from within this complex viral swarm [1]. This appears to be true for transmission mediated by all modes, including sexual transmission, mother-to-child transmission, and intravenous infection via needle sharing [1–9]. The virus populations in newly infected individuals often start off as genetically homogenous and match a strong consensus sequence during the first days and weeks after transmission [3,9]. Only over time does viral diversity begin to bloom. The critical question is whether the virions that seed and establish new infections possess unique biological properties or whether transmission is simply so inefficient that it produces an extreme (but random) genetic bottleneck. Indeed, recent studies show that virions isolated from early in infection do have some unique biological properties, and this is still an active area of research [3,5,10–15]. If it continues to be substantiated that early isolates have unique properties, prophylactic vaccines will only need to overcome this special subset of HIV-1, curtailing the significant problem that global viral diversity presents to vaccine design. This simplification of the problem has fueled new hope for the long-elusive HIV-1 vaccine.

**Fig 1:**
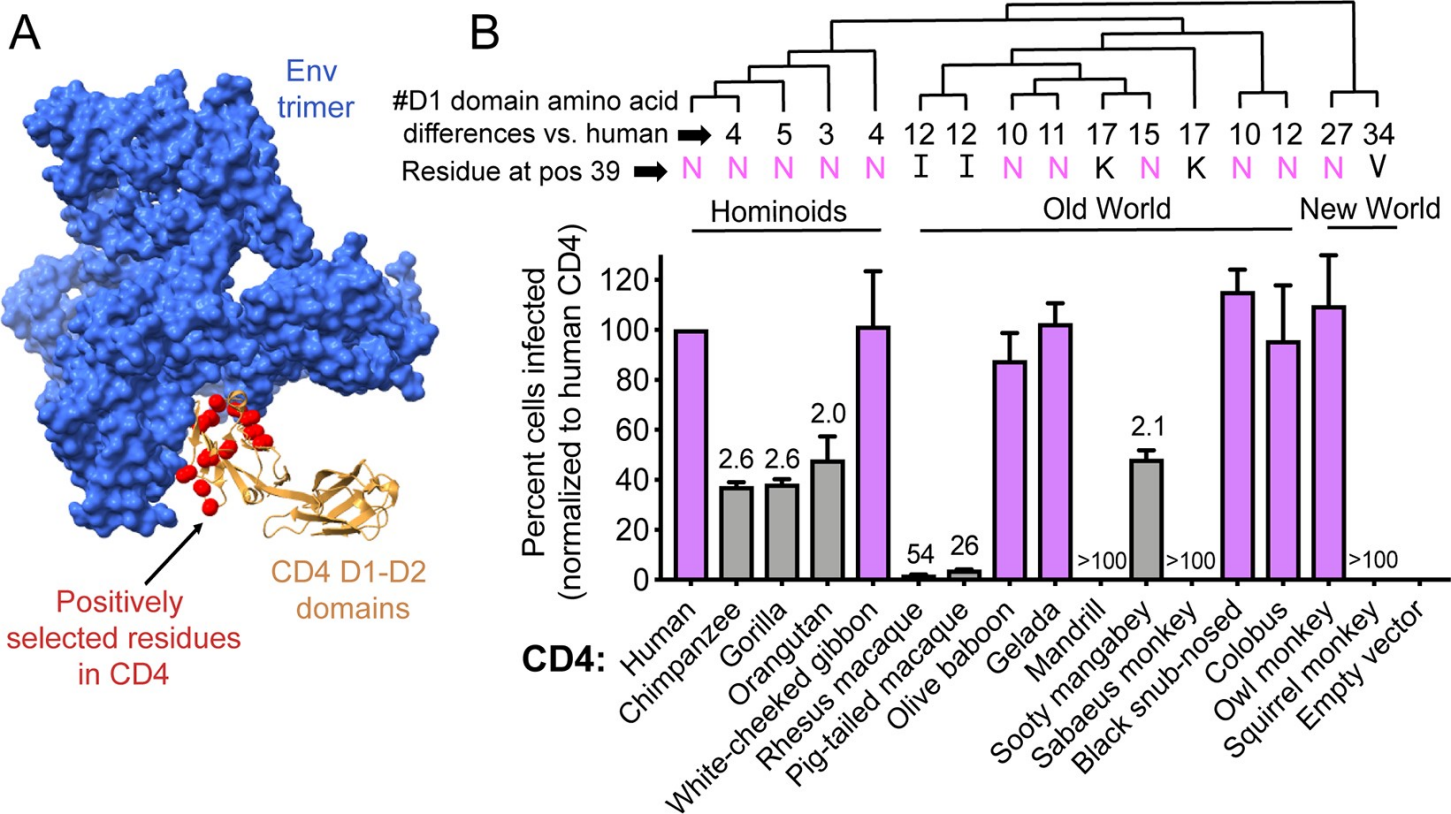
Some primate species encode a CD4 that functions as an entry receptor for an early isolate of HIV-1. (A) Cryo-EM structure of the Envelope (Env) trimer (blue) from a prototypic early HIV-1 isolate (BG505 [9]) in complex with human CD4 (tan) (PDB:5U1F) [28]. Only the D1 and D2 domains of CD4 are included in this structure, and the D1 domain mediates interaction with Env. Amino acids highlighted in red spheres were previously shown to be evolving under recurrent positive selection and fall at the interaction interface with Env [20,21]. (B) Dog thymocytes (Cf2Th cells) stably expressing human CCR5 and the indicated CD4 receptors (x-axis) were infected with Q23ΔEnv-GFP pseudotyped with BG505 Env, isolated from a newly infected infant [9]. The percent cells infected (GFP-positive) was enumerated by flow cytometry and normalized to the percent infected with human CD4. Error bars represent the mean + SEM from four independent experiments, each with two to three technical replicates. Values above error bars represent fold decrease in viral entry relative to human CD4, only indicated for those samples that passed significance thresholds (one-way ANOVA for repeated measures effect, Dunnett’s test; *P* < 0.05). Data associated with this figure can be found in the supplemental data file (S1 Data). (Top) Cladogram of species used in this experiment, with the number of amino acid differences in the D1 domain of CD4 (compared to human CD4) noted at the branch tips as well as the amino acid encoded at position 39. CCR5, C-C motif chemokine receptor 5; cryo-EM, cryogenic electron microscopy; GFP, green fluorescent protein; PDB, Protein Data Bank.

Primates have played a critical role in the history of HIV-1 [16]. HIV-1 originated from the zoonotic transmission of a simian immunodeficiency virus (SIV) to humans [17], and HIV-1 disease and transmission is now modeled in primates in the lab [18]. The natural reservoir of SIV in African primates is ancient, with over 40 primate species being endemically infected with unique SIVs [17,19]. It has been proposed that the long-term coevolutionary history between primates and SIVs may have affected the evolutionary trajectory of the CD4 gene [20,21], which encodes the main cell surface receptor for both HIV and SIV. The CD4 gene has experienced numerous rounds of natural selection in favor of new allelic versions, specifically favoring sequence change at the virus-binding surface of the molecule [20,21]. The most rapidly evolving residues in CD4 fall at the interaction interface with the HIV-1 surface protein Envelope (Env) (Fig 1A). One model to explain this evolutionary signature is that primate individuals harboring CD4 alleles resistant to SIV infection have a selectable advantage compared to their peers. While SIV can probably mutate to bypass most receptor blocks, there will not be significant pressure to do so until a substantial number of hosts harbor CD4 alleles resistant to SIV infection. In this way, evolutionary arms races can unfold even though hosts and viruses have starkly different evolutionary rates (for a review on this topic, see [27]). Indeed, we and others have observed that many host receptors have evolved under intense selective pressure to modify virus–host interaction interfaces [16, 22–26]. Despite speculation regarding the significance of the unusual evolutionary signatures in CD4 [20,21], a comprehensive functional comparison of CD4 from different primate species has not been conducted since an appreciation has been gained for the special properties of early HIV-1 isolates.

Here, we evaluate the ability of primate CD4 proteins to function as receptors for various classes of HIV-1. We are particularly interested in primary HIV-1 isolates derived from the blood of newly infected people, in light of the new appreciation for the special properties that relate to transmission [29]. One primate genus (macaques; both rhesus and pig-tailed species) has previously been evaluated for the ability of its CD4 receptor to support entry of early HIV-1 isolates. Unfortunately, the macaque CD4 receptor does not support entry of the vast majority of early HIV-1 isolates, frustrating attempts to model transmission and early HIV-1 infection in these animals [30–32]. On the other hand, we found that most allelic forms of owl monkey CD4 do support entry of early HIV-1 isolates [33]. Beyond these species, the compatibility of early HIV-1 isolates with primate CD4 receptors has not been explored. We reasoned that HIV-1 compatibility with primate CD4 receptors might be dynamic, given that CD4 is highly diverged in its protein sequence at the interaction interface with HIV-1 Env (Fig 1A).

## Results

### Primate CD4 receptors are functionally diverse in supporting HIV-1 entry

We began by cloning the CD4 genes of 15 primate species, plus human CD4. Using retroviral transduction, we stably introduced these genes into Cf2Th cells (dog thymocytes) that had also been engineered to express the human C-C motif chemokine receptor 5 (CCR5) coreceptor for HIV-1 entry (S1 Fig). Expression levels of these CD4 receptors on Cf2Th cells were similar to endogenous expression levels of CD4 seen on human immortalized T cells and on primate primary T cells that we isolated directly from blood (S1 Fig). Our goal was then to infect these cells with an early HIV-1 isolate. For the sake of this study, we refer to all viruses or Env clones that were isolated <150 days post infection as “early” and those isolated >150 days post infection as “chronic” isolates. This nomenclature is consistent with the Fiebig scale classification, for which Fiebig stages I–V (typically lasting until 70 to 150 days post infection) capture early infection stages and Fiebig stage VI indicates progression into chronic infection [34,35]. This is also consistent with other studies on transmitted or early HIV-1, which have used viruses isolated from human blood weeks to months after infection [5,9,31,36,37]. The properties of all of the HIV-1 Env isolates used in this study are summarized in S1 Table.

The HIV-1 Envelope clone “BG505” has risen to prominence in the study of early HIV-1. The Overbaugh group amplified BG505 Env from an infant approximately 6 weeks after delivery and showed that this Env sequence was nearly identical to the two other Env sequences amplified from this baby at the same time point [9]. This indicated that the infection probably started from a single virion and that the captured Env sequence closely resembles the Env of this transmitted virion. A cocrystal of BG505 Env has been solved in complex with CD4 (Fig 1A) [28,38–40], and the alternate folding conformations of BG505 Env have been characterized in depth [41]. We prepared HIV-1 pseudotyped with the BG505 Env by cotransfecting 293T cells with two plasmids: one expressing the BG505 Env, and another expressing an HIV-1 green fluorescent protein (GFP) reporter virus genome (Q23ΔEnv-GFP, representing a blood-derived clade A isolate from 1 year after seroconversion [30,42]). Cell lines expressing human CCR5 and different primate CD4 receptors were then infected with this BG505 virus (similar infection results were obtained in cell lines expressing matched CD4 and CCR5 from each species; S2 Fig). Forty-eight hours post infection, cells were harvested and analyzed by flow cytometry. Samples were first gated for live cells and further gated such that all samples were narrowed to the same log decade of CD4 receptor expression (S1 Fig). GFP-positive (i.e., infected) cells were then enumerated within this population. We identified three categories of CD4 receptors that support HIV-1 entry to varying degrees (Fig 1B). The CD4 receptors of some species (shown with purple bars: white-cheeked gibbon, olive baboon, gelada, black snub-nosed monkey, colobus, and owl monkey) behave similarly to human CD4 when challenged with BG505 HIV-1. These species are not our closest relatives but rather are distributed throughout the primate phylogeny (Fig 1B). A second class of species (shown with gray bars: chimpanzee, gorilla, orangutan, and sooty mangabey) encodes CD4 receptors that support entry of this virus but at a level that is approximately 2-fold lower than human CD4. A third class of species (macaques, mandrills, sabaeus monkeys, and squirrel monkeys) encodes CD4 receptors that support entry at a level 25-fold or more reduced compared to human CD4. With some of the CD4 receptors in the latter class, infection was reduced by more than 100-fold compared to human CD4. While the chimpanzee CD4 allele tested here has a semi-permissive phenotype, we have recently shown that some chimpanzee CD4 alleles are highly resistant to HIV-1 entry [48]. As a control, we determined that infection did not change the surface expression level of human or any primate CD4 receptor (S1 Fig). This allows us to conclude that the observed differences in infection are due to defects in entry and that our gating strategy did not exclude infected cells because they no longer express CD4. From this data, we can conclude that CD4 receptors are functionally variable from one primate species to the next with regards to supporting entry of an early HIV-1 isolate. Prior to this, there was only one primate species known to encode a CD4 receptor compatible with early HIV-1 isolates, owl monkeys [33], but now we can add five additional species to that list: white-cheeked gibbon, olive baboon, gelada, black snub-nosed monkey, and colobus monkey.

HIV-1 entry requires engagement of the amino-terminal “D1” domain of the CD4 receptor (residues 1–98). This is evidenced by numerous Env–CD4 cocrystals [43–45] and mapping of D1-domain residues that affect interaction with HIV-1 Env [43,46–48]. For instance, the macaque CD4 receptor can be rendered functional for HIV-1 entry by introducing a single amino acid substitution in the D1 domain (isoleucine “39I” mutated to the human residue, asparagine “39N”) [31]. Likewise, owl monkey populations circulate CD4 alleles that are both non-functional (39I) and functional (39N) for HIV-1 entry [33]. From the panel of primate CD4s that we have tested, we can now build on this observation that residue 39 in CD4 is particularly important for HIV-1 engagement. In Fig 1B, the residues encoded at CD4 position 39 in each species are listed at the tips of the phylogeny, along with the total number of amino acid differences in the D1 domain compared to human CD4. From this, it appears that CD4 must encode an asparagine (“N”) at position 39 in order to have any appreciable function as a receptor for BG505 HIV-1. Indeed, position 39 in CD4 has experienced positive selection during primate speciation [20,21], and this may be because mutations that replace the asparagine protect individuals against infection. However, there are clearly sequence determinants outside of position 39 that also matter because not all receptors with an asparagine at position 39 function equally well.

### The selective use of primate CD4 receptors is a property of HIV-1 from the bloodstream, including early HIV-1 isolates

**Fig 2:**
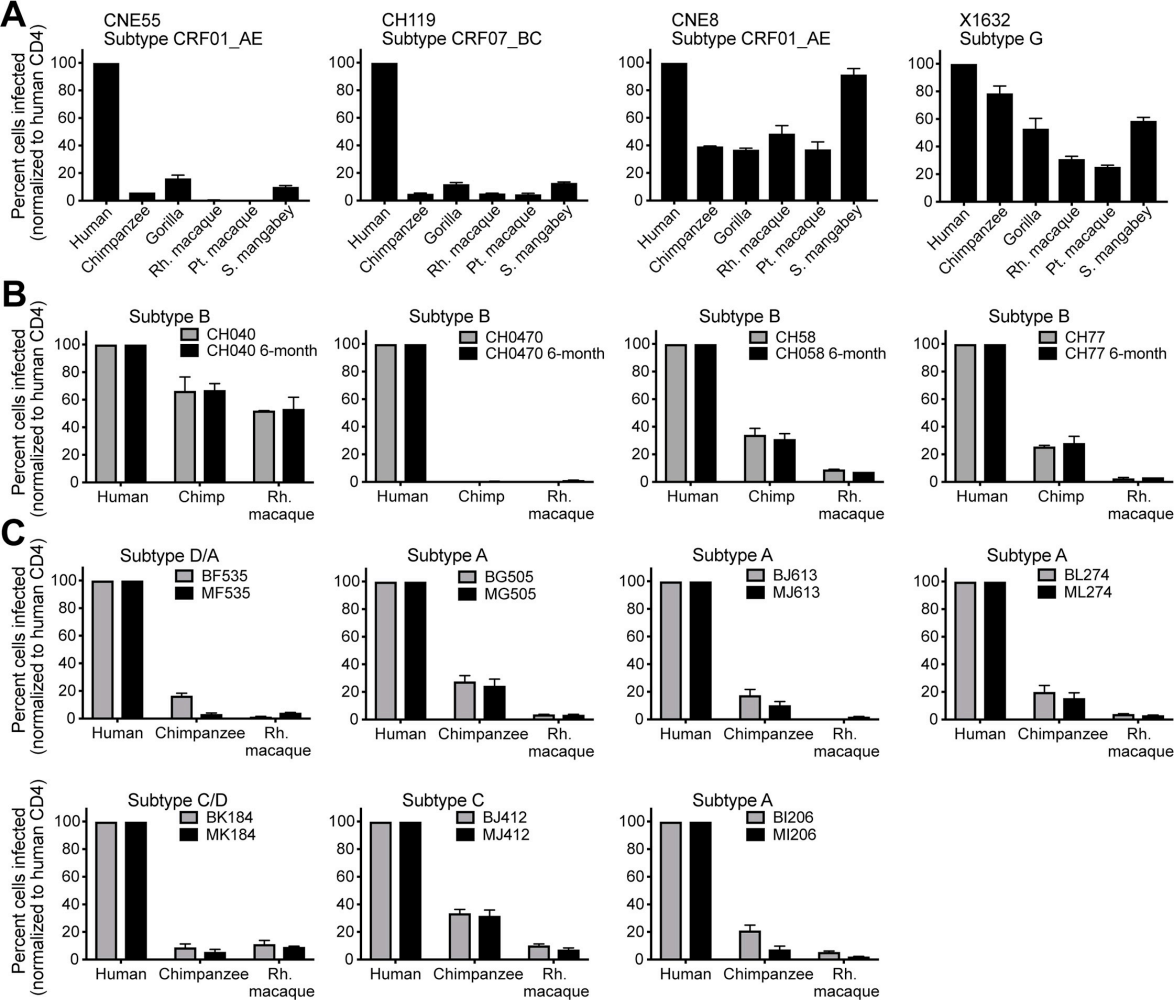
Selective use of primate CD4 receptors is not a result of the transmission bottleneck and instead is seen in many isolates of HIV-1 taken from human blood. Cells stably expressing human CCR5 and human or primate CD4 (x-axis) were infected with Q23ΔEnv-GFP bearing (A) chronic Envelopes (Envs) [49], (B) early (newly infected; grey bars) and chronic (6 months post infection; black bars) Env pairs derived from the same patient [3,14,50,51], shown for one patient in each panel, or (C) Envs derived from maternal (chronic; black bars)/infant (newly infected; grey bars) transmission pairs [9], with one mother–baby pair shown in each panel. The percent cells infected (GFP-positive) was measured by flow cytometry and normalized to the percent infected with human CD4. Error bars represent the mean + SEM from two independent experiments, each with two to three technical replicates. Data associated with this figure can be found in the supplemental data file (S2 Data). CCR5, C-C motif chemokine receptor 5; GFP, green fluorescent protein.

We next sought to determine whether the selective use of only some primate CD4 receptors is a property specific to early HIV-1 isolates. From this point forward, we focused on CD4 receptors from a subset of primate species, in particular the African primate species involved in the zoonotic transmissions of HIV-1 (chimpanzees and gorillas) and HIV-2 (sooty mangabeys) [17], and the macaque species that serve as the current laboratory model for HIV-1 (expression histograms for these cell lines are shown in S2 Fig). All of these receptors were partially or highly defective for entry mediated by BG505 Env (Fig 1B). We next tested Envs isolated from the blood of four individuals chronically infected with HIV-1 at time points more than 6 months after the original infection [49]. These Envs were pseudotyped onto Q23ΔEnv-GFP, as described above. These viruses were then used to infect stable cell lines expressing human CCR5 and CD4 from different primate species. Two of the four Envs demonstrated the specific use of human CD4 that we have described (Fig 2A, left two panels). The other two demonstrated a weakening of this selective CD4 phenotype but still a preference for human CD4 (Fig 2A, right two panels). This suggests that the phenotype of selective CD4 usage may be retained during later stages of disease and may not be unique to early HIV-1 isolates.

To address this further, we looked at how CD4 receptor usage may change over time as infection progresses from early- to late-stages of disease. To test this, we made use of sets of molecular clones that had been amplified by single-genome amplification from patient blood early after infection and then again from the same patient 6 months later [3,14,50,51]. From four such sets of molecular clones, we cloned the rev-env cassettes into a mammalian expression vector, which was then used to pseudotype each Env onto Q23ΔEnv-GFP, as described above. These viruses were tested for their ability to enter cell lines stably expressing human or primate CD4 receptors, along with human CCR5. We observed no significant difference in receptor usage between the early Env (grey bars) and its matched 6-month counterpart (black bars) (Fig 2B). In fact, for each patient, the Envs derived from the two different time points (6 months apart) behaved almost identically. Infection mediated by all of these Envs was reduced in cells expressing chimpanzee or macaque CD4 receptors when compared to human CD4. One exception was seen with virus from patient CH40, whose Env did mediate entry somewhat better via chimpanzee and macaque CD4 compared to other Envs, although still less well than with human CD4. Therefore, selective use of primate CD4 receptors appears to describe many HIV-1 isolates from the blood, including those isolated just after infection (weeks), later at 6 months (Fig 2B), or even during chronic stages of infection (Fig 2A).

**Fig 3:**
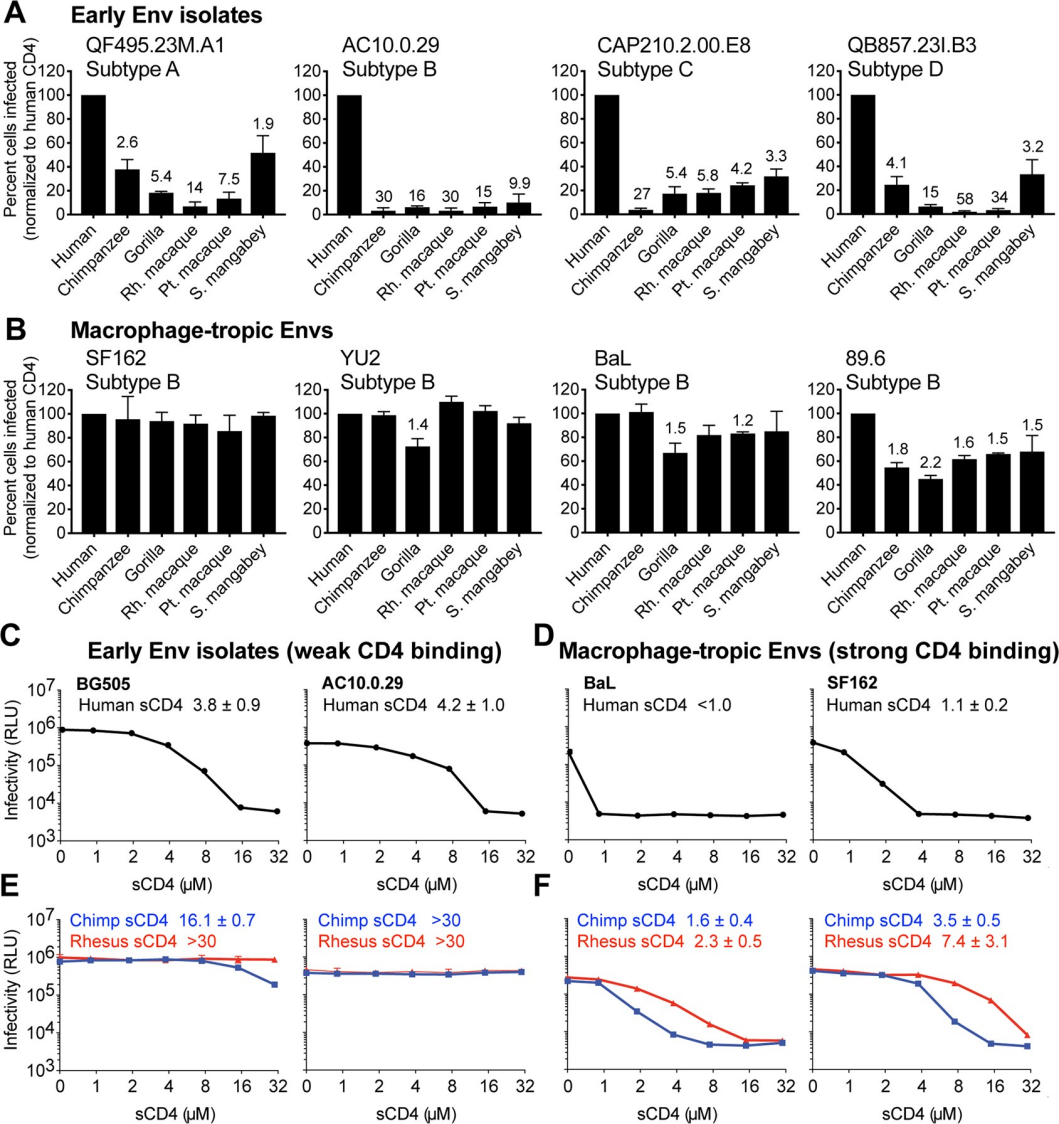
Macrophage-tropic HIV-1 isolates, which are known to bind human CD4 more tightly, are promiscuous in their use of primate CD4 receptors. (A, B) Cells stably expressing various primate CD4 receptors (x-axis), along with human CCR5, were infected with Q23ΔEnv-GFP pseudotyped with (A) early HIV-1 Envelopes (Envs) or (B) Envs from common macrophage-tropic HIV-1 isolates. The percent cells infected (GFP-positive) was measured by flow cytometry and normalized to the percent infected with human CD4. Error bars represent the mean + SEM from two independent experiments, each with two to three technical replicates. Values above error bars represent fold decrease in viral entry relative to human CD4, only indicated for those samples that passed significance thresholds (one-way ANOVA for repeated measures effect, Dunnett’s test; *P* < 0.05). (C and D) Infection of TZM-bl cells with Q23ΔEnv-GFP pseudotyped with early (C) or macrophage-tropic (D) Envs. Each virus was preincubated with increasing concentrations of human soluble CD4 (sCD4) as a competitive inhibitor. TZM-bl cells are HeLa CD4/CCR5 cells that express luciferase in response to HIV-1 tat expression, read in relative light units (RLUs). Error bars represent the mean + SD from one biological replicate (*n* = 3 to 4 technical replicates), representative of two independent experiments. Error bars are not plotted in instances in which the size of the error bar is less than the symbol size. IC_50_ values from the soluble CD4 neutralization assays are shown within each panel, for which the mean and SD values reflect the variation of the IC_50_ calculations from two independent experiments. (E and F) Same as C and D, only neutralization assays were performed with chimpanzee and rhesus macaque sCD4 instead of human sCD4. Data associated with this figure can be found in the supplemental data file (S3 Data). CCR5, C-C motif chemokine receptor 5; GFP, green fluorescent protein.

We next examined the transmission bottleneck directly by testing matched donor–recipient HIV-1 Env pairs. HIV-1 was pseudotyped with Envs from mother–infant pairs, of which the mother was chronically infected and her baby was newly infected (<6 weeks) [9]. For seven such pairs, we found that mother HIV-1 Envs (black bars) were not different from infant HIV-1 Envs (grey bars), in that all demonstrated the selective use of human CD4 (Fig 2C). In fact, corresponding mother and baby Envs behaved very similarly. When the seven pairs were grouped, no significant differences were noted between maternal (chronic) and infant (early) Envs in terms of their ability to infect cells bearing chimpanzee or rhesus macaque CD4 receptors (Mann–Whitney test, *P* > 0.05). The tested pairs represented most major HIV-1 group M subtypes, suggesting that selective use of CD4 may describe all or most globally circulating forms of HIV-1. We next verified this by challenging cell lines expressing different CD4 receptors with early HIV-1 isolates representing each of the four major group M (pandemic) HIV-1 subtypes. These four Envs, isolated from patient blood shortly after initial infection [36,52,53], were pseudotyped onto the Q23ΔEnv-GFP virus, as described before. While human CD4 supported entry of all of these viruses, primate CD4 orthologs supported levels of HIV-1 entry that ranged from 2- to 58-fold lower (Fig 3A). Collectively, our study has explored a breadth of HIV-1 isolates taken from patient blood, representing all of the major subtypes found globally, and has found that they can all be phenotypically described by their selective use of only certain primate CD4 receptors.

### Selective use of only certain primate CD4 receptors results from weak CD4 binding affinity

Next, we wanted to understand more about why blood-derived HIV-1 Env isolates demonstrate this selective use of only some primate CD4 receptors. Thus far, all of the Envs that we have tested are from CCR5-tropic viruses isolated from the blood. There are three types of HIV-1 in the body of an infected person: CCR5 T cell-tropic (the most abundant form in the blood, utilizing the CCR5 coreceptor), CXCR4 T cell-tropic (in the blood but using an alternate coreceptor, C-X-C chemokine receptor type 4 [CXCR4]), and CCR5 macrophage-tropic (rare in the blood, usually found in the central nervous system) [29,54–56]. Only the first of these transmits to new individuals, whereas the latter two types arise in special evolutionary niches within the human body during the course of chronic infection and rarely transmit [57–63]. In this study, we did not consider or test CXCR4-utilizing viruses. We next tested HIV-1 pseudotyped with Envs from four macrophage-tropic viruses [31,64–72] for their ability to infect cells bearing primate CD4 receptors (Fig 3B). In stark contrast to the blood-derived isolates tested above (Figs 1, 2 and 3A), macrophage-tropic viruses were promiscuous in their use of all CD4 receptors tested. For each primate CD4 tested, the median relative infection for all early HIV-1 Envs (Fig 3A) was significantly lower than macrophage-tropic Envs (Fig 3B; *P* < 0.05; Mann–Whitney U test).

Macrophage-tropic HIV-1 Env is known to bind CD4 with higher affinity compared to other forms of HIV-1 Env [73–75]. This is a selected property that these virions possess because macrophages have lower densities of CD4 molecules on their surface [74]. Based on this, we hypothesized that weak CD4 binding affinity is what makes most blood-derived HIV-1 Envs sensitive to small sequence differences in CD4 receptors from different primates. To test this, we first purified a soluble version of the human CD4 receptor for use in neutralization assays (sCD4, consisting of the D1 and D2 domains of CD4) (S3 Fig). When sCD4 is preincubated with HIV-1, it is known to competitively inhibit virus interaction with the CD4 expressed on the cell surface and block virus entry [76]. Our purified human sCD4 behaved identically to a commercially available version (S3 Fig). HIV-1 pseudotyped with early or macrophage-tropic Envs was preincubated with increasing concentrations of sCD4 and then used to infect TZM-bl indicator cells (HeLa CD4/CCR5 cells that express luciferase in response to HIV-1 tat expression). HIV-1 pseudotyped with either of two different early Envs (BG505 or AC10.0.29) was neutralized in a dose-dependent manner by human sCD4 (Fig 3C). As expected, HIV-1 pseudotyped with either of two macrophage-tropic Envs (BaL or SF162) was neutralized by human sCD4 to an even greater extent, consistent with these viruses having a higher affinity for CD4 (Fig 3D). The IC_50_ values are shown within each panel and are approximately 4-fold higher for early Envs than for macrophage-tropic Envs. In conclusion, the broadened ability to enter cells through the CD4 receptors encoded by all primates tested (Fig 3A and 3B) correlates with tighter CD4 binding (Fig 3C and 3D).

We next wanted to confirm that weak binding to human CD4 receptors results in a loss of binding to primate CD4 receptors. We purified chimpanzee and rhesus macaque sCD4 (S3 Fig) and tested the ability of these proteins to neutralize different classes of HIV-1. Viruses bearing macrophage-tropic Envs (BaL or SF162) were neutralized by chimpanzee and macaque sCD4 (Fig 3F), while viruses bearing Envs from early HIV-1 isolates (BG505 or AC10.0.29) were not neutralized to an appreciable degree (Fig 3E). Collectively, these data suggest that most early HIV-1 isolates from the blood, which have lower affinity for human CD4 compared to macrophage-tropic viruses, do not bind well to chimpanzee and macaque CD4. This shows that our entry assay for selective versus promiscuous use of primate CD4s is essentially a readout of CD4 binding affinity.

**Fig 4:**
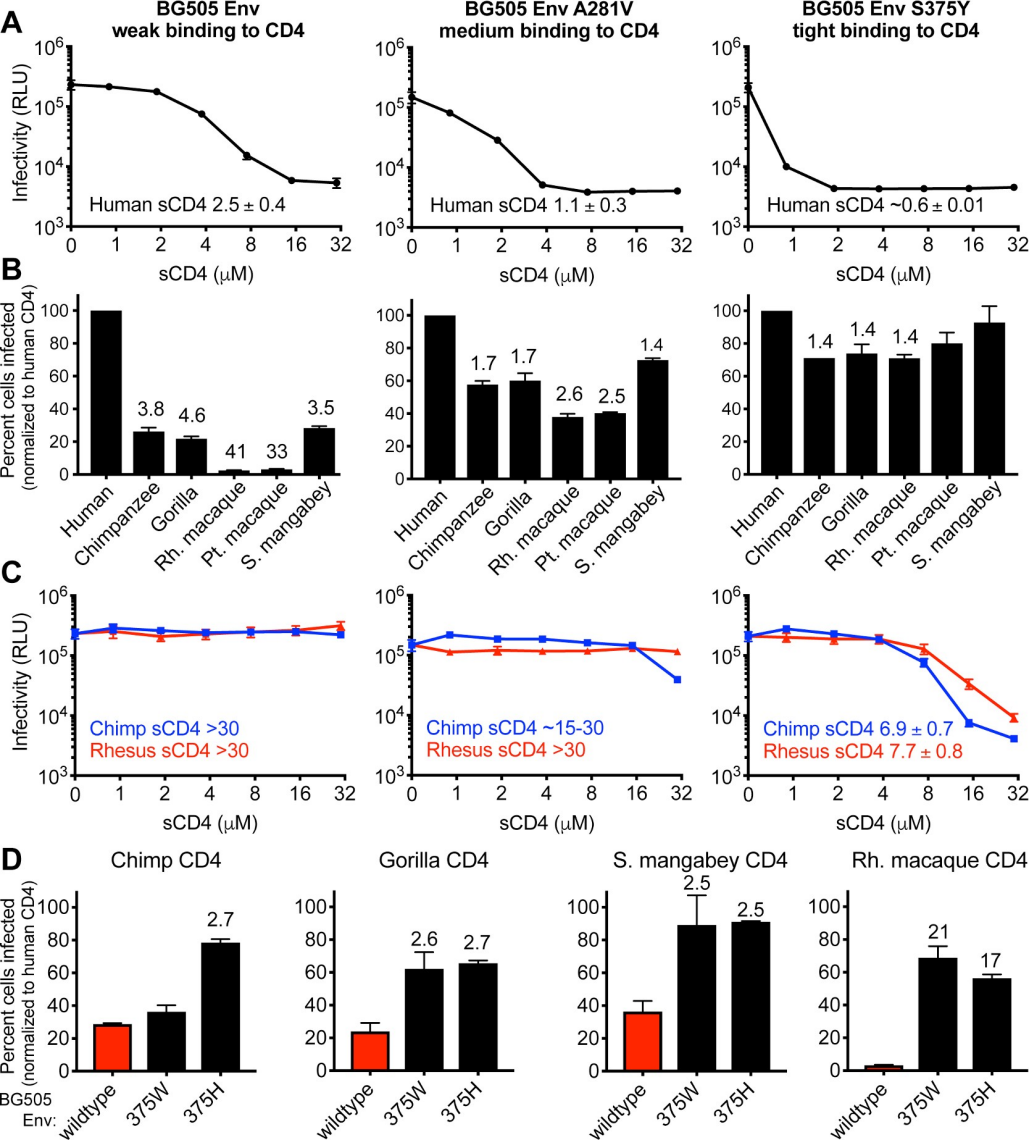
An early Env engineered to have weak, medium, or tight binding to CD4 becomes progressively more promiscuous in its use of primate CD4 receptors. (A–C) Amino acid substitutions were introduced into the BG505 Envelope (Env) at position 281 and 375. The headers at the top describing these mutations pertain to panels A, B, and C. In each column, the indicted Env was pseudotyped onto Q23ΔEnv-GFP. (A) Each of the three viruses was preincubated with increasing concentrations of human soluble CD4 (sCD4) and then used to infect TZM-bl cells, which are HeLa CD4/CCR5 cells that express luciferase in response to HIV-1 infection and tat expression, read in relative light units (RLUs). Error bars represent the mean + SD from one biological replicate, representative of two independent experiments. Error bars are not plotted in instances in which the size of the error bar is less than the symbol size. IC_50_ values are listed on each panel, for which the mean and SD values reflect the variation of the IC_50_ calculations from two independent experiments. (B) Cells stably expressing the indicated CD4s (x-axis) and human CCR5 were infected with each virus. The percent cells infected (GFP-positive) was measured by flow cytometry and normalized to the percent infected with human CD4. Error bars represent the mean + SEM from two independent experiments, each with three technical replicates. Values above error bars represent fold decrease relative to human CD4 for those samples that passed significance thresholds (one-way ANOVA for repeated measures effect, Dunnett’s test; *P* < 0.05). (C) Same as panel A but with chimpanzee and rhesus macaque sCD4 instead of human sCD4. (D) Two additional single point mutations were introduced into the BG505 Env, which have also been shown to increase binding affinity for CD4 [77]. These Envs were pseudotyped onto Q23ΔEnv-GFP and used to infect cells stably expressing the indicated CD4 (top of each graph) and human CCR5. The percent cells infected (GFP-positive) was measured by flow cytometry and normalized to the percent infected with human CD4. Error bars represent the mean + SEM from two independent experiments, each with two to three technical replicates. Values above error bars represent fold increase relative to wildtype BG505 Env for those samples that passed significance thresholds (one-way ANOVA for repeated measures effect, Dunnett’s test; *P* < 0.01). Data associated with this figure can be found in the supplemental data file (S4 Data). CCR5, C-C motif chemokine receptor 5; GFP, green fluorescent protein.

To further test this premise, we next created a panel of Envs engineered to have high, medium, or low binding affinity for CD4. BG505 Env, the prototypic early Env discussed above, was engineered to have two different point mutations, one at position 281 and the other at position 375. These mutations in Env have been previously shown to increase binding affinity to CD4, although only one of them has been characterized within the BG505 Env background [77,78]. Consistent with these previous reports, the A281V mutation subtly increased binding to human sCD4 relative to wild type (demonstrated by increased neutralization by sCD4), while the S375Y mutation resulted in a more substantial increase (Fig 4A). While both of these mutations were originally characterized because they increase entry via macaque CD4 [77,78], we found that both mutations also improve entry via all of the primate CD4 receptors tested, with increasing improvement as the binding affinity for CD4 increases (left to right in Fig 4B). This pattern of increased binding affinity for human CD4 and increased ability to enter cells via primate CD4s also correlated to increased binding affinity for primate CD4 receptors, as read in a neutralization assay with purified chimpanzee and rhesus macaque sCD4 proteins (left to right in Fig 4C). Finally, we engineered two additional mutations into HIV-1 BG505 Env that have been shown to increase CD4 binding and to increase entry via macaque CD4 [77]. These mutations also make Env able to more efficiently mediate entry via all primate CD4s tested, not just that of macaques (Fig 4D). These data support the general premise that promiscuous use of primate CD4s can be created by mutations in Env that increase CD4 binding affinity. Further, the promiscuous use of primate CD4 receptors is not just a property of macrophage-tropic viruses but instead may be a common property of viruses engineered or adapted to bind CD4 more tightly. The fact that virtually no blood-derived HIV-1 isolate that we have tested has this phenotype (promiscuous use of primate CD4 receptors) suggests that this phenotype is strongly selected against in the human body. The reasons for this are unknown.

**Fig 5:**
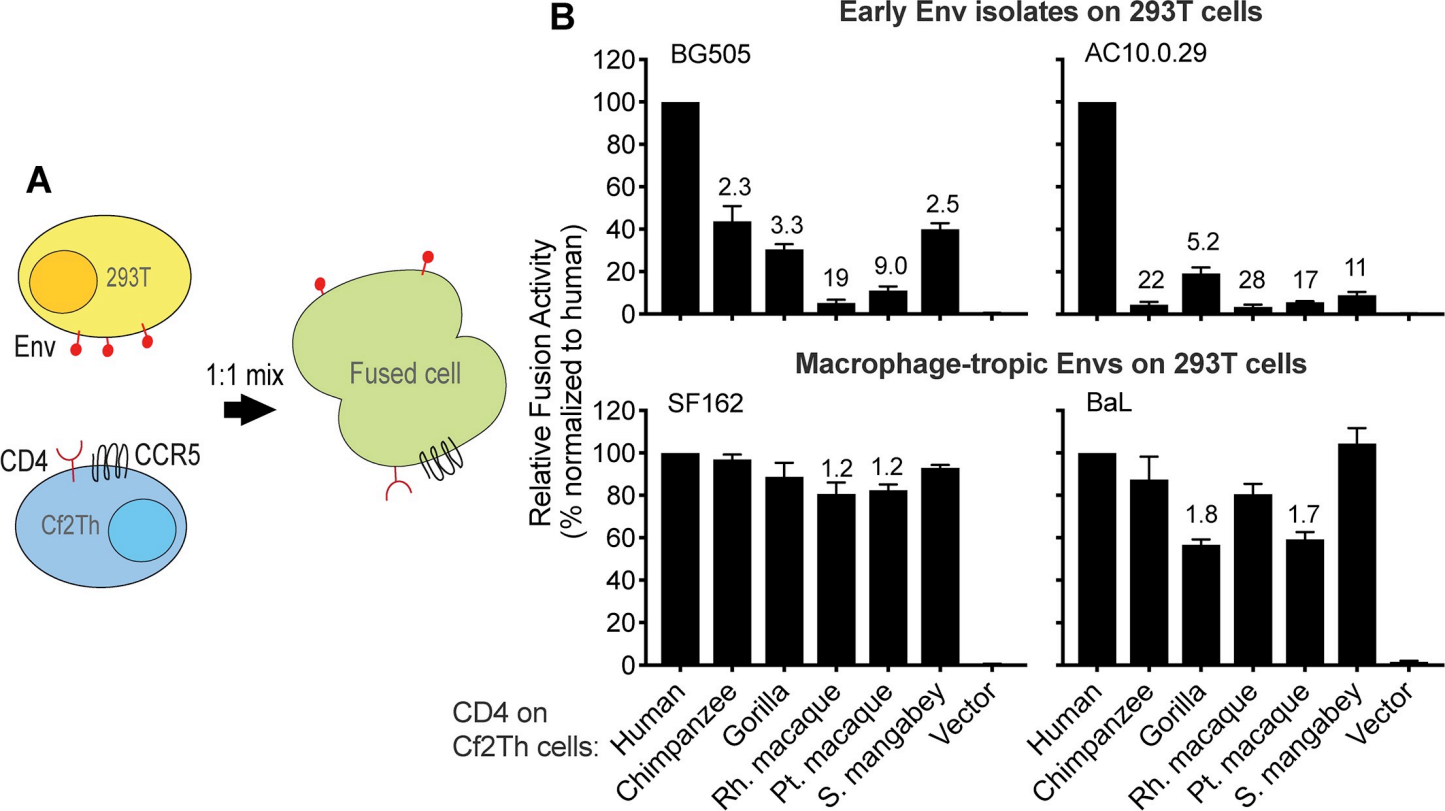
Early isolates of HIV-1 from the blood are deficient in binding and/or fusion with CD4 from some primate species. (A) 293T cells transiently expressing various Envelope proteins (Envs), and dog thymocytes (Cf2Th cells) stably expressing various primate CD4 receptors and human CCR5, were each transfected with plasmids encoding half of a split Renilla luciferase [79]. These two cell types were then cocultured to induce membrane fusion, which was detected by a luminescent product following the addition of the Renilla luciferase substrate. (B) The percentage of fusion was calculated relative to what was observed with human CD4. Error bars represent the mean + SEM from three to four independent experiments, each with four technical replicates. Values above error bars represent fold decrease relative to human CD4 for those samples that passed significance thresholds (one-way ANOVA for repeated measures effect, Dunnett’s test; *P* < 0.05). Data associated with this figure can be found in the supplemental data file (S5 Data). CCR5, C-C motif chemokine receptor 5.

We then employed a virus-free approach to confirm that the mechanism of selective use of certain primate CD4 receptors stems from weak CD4 binding, as opposed to other interactions between HIV-1 and CD4 that may occur during the course of an infection. Cells expressing HIV-1 Env on their surface were mixed with cells expressing human CCR5 and various primate CD4 receptors (Fig 5A). Cellular fusion was assessed using a split luciferase reporter system in which, upon cell–cell fusion, the two halves of luciferase merge and produce a functional enzyme [79]. We used this assay to test the interactions between four different Envs and six different CD4 receptors, as shown in Fig 5B. In this system, the phenotypes seen in our previous infection assays were largely recapitulated despite the fact that this assay is virus-free and only requires three proteins (Env and CD4/CCR5). The early Envs mediated fusion most efficiently with cells expressing human CD4, while the macrophage-tropic Envs broadly engaged and fused with diverse primate CD4 receptors. This assay, when taken together with the experiments employing purified CD4 described above, reveal that weak binding to CD4 is what makes most blood-derived HIV-1 isolates (i.e., those fueling the global pandemic) selective in their use of only certain primate CD4 receptors.

### Early HIV-1 isolates are blocked from entry by multiple allelic versions of macaque CD4

**Fig 6:**
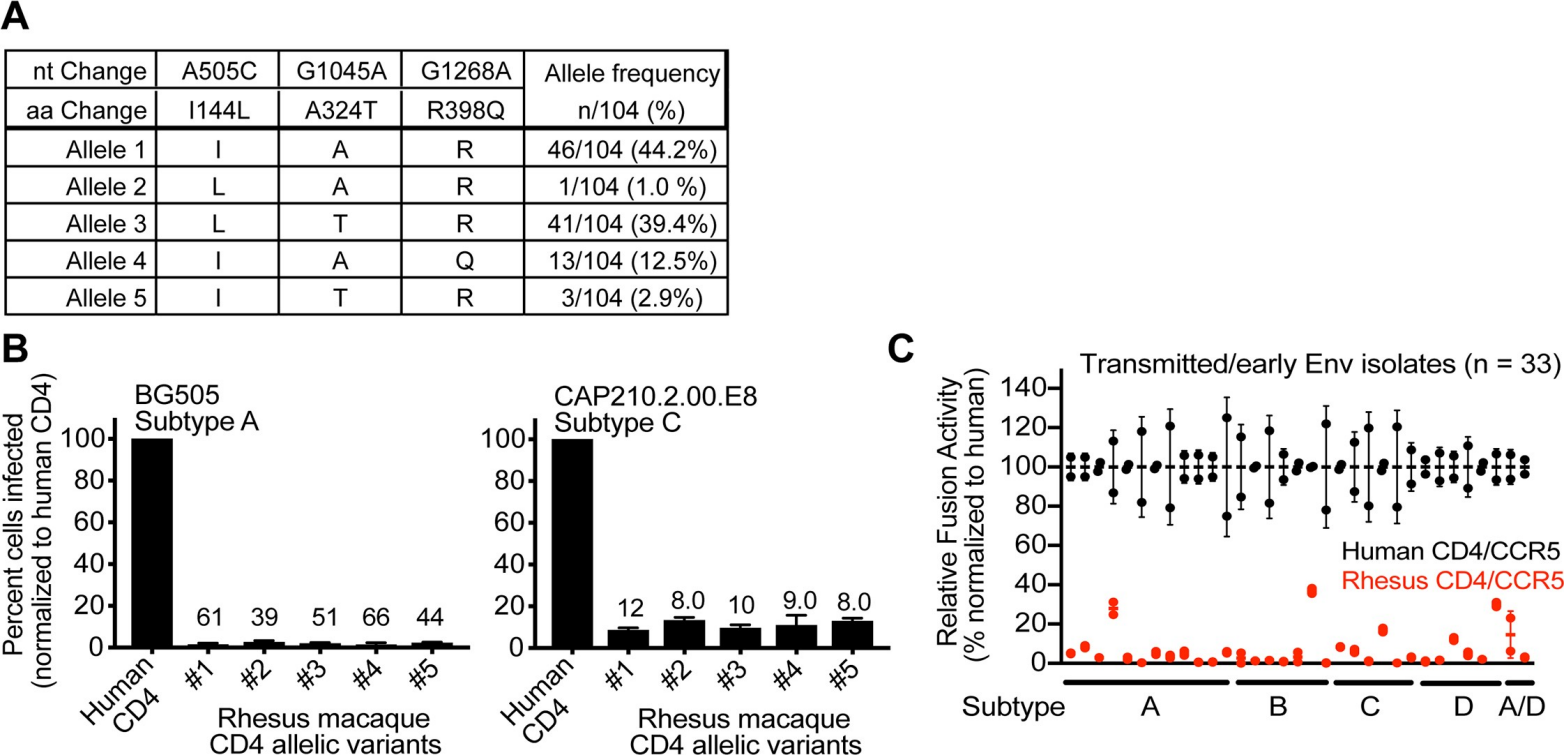
Rhesus macaque CD4 alleles are universally defective for HIV-1 entry. (A) Total RNA was isolated from the blood of 52 rhesus macaque individuals. CD4 was amplified from cDNA, and PCR products were sequenced to yield genotype information for each individual (104 chromosomes). Genotype information was used to phase SNPs using Phase 2.1 [81,82] in DNAsp v5 [83]. The table summarizes the unique CD4 alleles identified. For each allele, the residue encoded at each variable amino acid positions is noted. Unique alleles were cloned and further verified by Sanger sequencing. (B) Dog thymocytes (Cf2Th cells) stably expressing human CCR5 and the indicated rhesus macaque CD4 alleles (x-axis) were infected with HIV-1 (Q23ΔEnv-GFP) bearing a subtype A (BG505) or subtype C (CAP210.2.00.E8) Envelope (Env). The percent cells infected (GFP-positive) was measured by flow cytometry 48 hours post infection. Error bars represent the mean + SEM from two independent experiments, each with three technical replicates. Values above error bars represent fold decrease relative to human CD4 for those samples that passed significance thresholds (one-way ANOVA for repeated measures effect, Dunnett’s test; *P* < 0.05). (C) 293T cells transiently expressing diverse HIV-1 Envs (see S1 Table) and Cf2Th cells stably expressing the indicated CD4/CCR5 receptors were each transfected with plasmids encoding half of a split Renilla luciferase [79]. These two cell types were then cocultured to induce membrane fusion, which was detected by a luminescent product following the addition of the renilla luciferase substrate. A total of 33 Envs were tested from the following major HIV-1 subtypes: subtype A (*n* = 12), subtype B (n = 7), subtype C (*n* = 6), subtype D (*n* = 6), and subtype A/D (*n* = 2). The percentage of fusion was calculated relative to what was observed with human CD4/CCR5. Error bars represent the mean + SD, for which *n* = 2 (two technical replicates from one experiment). Data associated with this figure can be found in the supplemental data file (S6 Data). nt, nucleotide; aa, amino acid; CCR5, C-C motif chemokine receptor 5; GFP, green fluorescent protein.

Interestingly, macaques (both rhesus and pig-tailed) encode a CD4 receptor that is not permissive for entry of most blood-derived HIV-1 isolates (Figs 1, 2, 3 and 4), consistent with previous reports [30,77,78]. Since macaques (predominantly rhesus macaques) serve as the current animal model for HIV-1, we sought to determine whether some allelic variants of rhesus macaque CD4 might encode receptors that better support HIV-1 infection. We sequenced the CD4 gene from 52 captive Indian-origin rhesus macaques and identified alleles encoding five unique CD4 protein variants (Fig 6A). All of these allelic versions of rhesus macaque CD4 behaved identically and were nonfunctional for entry of virus pseudotyped with BG505 Env and also with Env from another early HIV-1 isolate, CAP210.2.00.E8 (Fig 6B). Therefore, while owl monkey and chimpanzee populations circulate CD4 alleles with dramatically different functions as receptors for HIV/SIV entry [33,48,80], it appears that the function of macaque CD4 is identical from one allele to the next.

In this study, we have shown that Envs from over 30 early and chronic HIV-1 isolates from human blood (mother–infant pairs, etc.) all demonstrate selective entry via only some primate CD4 receptors. We next tested 29 additional Envs (*n* = 33 total, four of which were previously tested in infection assays herein; S1 Table) using the cell–cell fusion assay described above. These Envs were universally poor at mediating fusion with cells expressing rhesus macaque CD4 and CCR5 proteins (Fig 6C). Similar data have been generated in other large screens of early HIV-1 Envs [31,32]. Collectively, it seems that virtually no HIV-1 isolated from the bloodstream is compatible with the macaque version of CD4. If we now understand the inability of HIV-1 to use macaque CD4 to be a reflection of weak CD4 binding, this leads us to understand that virtually all early- and late-stage HIV-1 isolates in the human bloodstream experience continual selection to retain weak CD4 binding.

## Discussion

We find that many HIV-1 isolates from the blood, including early HIV-1 isolates, enter cells poorly through a subset of primate CD4 receptors. This finding is highly analogous to what has been observed with primate restriction factors that block HIV-1 infection, in that they too are highly species-specific in their interaction with HIV-1 [84,85]. Like CD4, most restriction factors are also evolving under intense positive selection in primates [86–89], explaining why these proteins vary in protein sequence on virus-binding surfaces from one host species to the next. It appears that SIVs, and probably other viruses as well, have put intense selective pressure on these genes to evolve in a way that changes the protein sequence on virus-binding surfaces. The selection and retention of new CD4 alleles that block virus entry could protect a species from infection, at least until the virus population counter adapts. When this type of arms race is playing out in multiple host species independently, it drives divergence in protein sequence at host–virus binding interfaces and has the long-term effect of making it difficult for viruses to move between species [16]. Indeed, a prevailing theme that has emerged in recent years is that receptor sequence divergence serves as a potent barrier to the movement of viruses between species [16,90–94]. Likewise, this study suggests that HIV-1 entry is blocked by the CD4 receptor of some primate species that it might encounter, whether that be in the wild or in the lab. On the other hand, we identify several primate species that encode CD4 receptors compatible with unmodified isolates of HIV-1 from the human bloodstream, including early isolates. These species could possibly be useful as animal models of HIV-1 transmission and infection, assuming that restriction factor blocks can be overcome in these species.

We show that selective use of only certain primate CD4 receptors occurs when HIV-1 Env has weak binding affinity for CD4. This weak affinity makes HIV-1 Env sensitive to sequence differences in the CD4 molecule. The strongest evidence in favor of this model is that viruses that have been selected or engineered to have tight CD4 binding affinity become agnostic to sequence differences in primate CD4 and can more efficiently enter cells using all CD4 orthologs that we have tested. While neutralization assays to assess the CD4 binding affinity of a given virus are laborious, our assay for selective or promiscuous primate receptor usage allowed us to screen a large number of HIV-1 isolates from human blood. Based on this, we show that weak CD4 binding is a nearly constant property of HIV-1 circulating in the human bloodstream, including but not specific to early-stage virions. This underscores the need to further understand the significance of CD4 binding affinity to HIV biology. While it has long been known that different HIV types have different affinities for CD4, the functional consequences of this are unclear. This is important to understand because weak CD4 binding seems to be selected for in the human bloodstream, yet some of our current primate models use Envs that are engineered for tight CD4 binding affinity so that they more efficiently engage the nonpermissive macaque CD4 receptor. For example, mutations at HIV-1 Env 375, when engineered into SHIVs, render virons compatible with macaque CD4 [77]. However, mutations at this position do not emerge in vivo when HIV-1 Envs are adapting to macaque CD4, whereas mutations at Env 281 do [78]. Based on our data, we can now speculate that Envs with high binding affinity to CD4 have a fitness penalty in vivo.

Given that nearly all HIV-1 in the human bloodstream seems to be selected to bind CD4 weakly, why would this be preferable? Although the reasons for this are currently unclear, there have been numerous speculations put forward [29]. It is possible that more efficient use of CD4 requires changes in Env conformation that would increase susceptibility to antibody neutralization. Indeed, Arrildt and colleagues have shown that when compared to patient matched T-cell tropic isolates from the blood, macrophage-tropic isolates appear to be more sensitive to certain CD4 binding site antibodies [95]. Thus, any conformational change that increases antibody neutralization sensitivity would likely be purged from the viral population. Alternatively, tighter CD4 binding is known to enhance HIV-1 tropism for cells of the monocyte–macrophage lineage [73,95,96]. However, replication of HIV-1 in these cells appears to be slowed due to cell type–specific blocks, including the restriction factors SAMHD1 and APOBEC3A [97,98], delayed reverse transcription and integration [99,100], and transcriptional repression [101]. These blocks to replication in macrophages may put high-affinity CD4 binders at an evolutionary disadvantage during early stages of infection. Finally, highly efficient macrophage infection may directly promote disease via inflammatory processes. HIV-1 infection of central nervous system macrophages coincides with severe inflammation and neuronal impairment [102–105]. Additionally, respiratory and cardiac dysfunction during late-stage HIV-1 infection has been associated with chronic inflammation, of which macrophages may be playing a central role [106,107]. Viral infection is a delicate balance between replicating efficiently enough to transmit, yet limiting disease to maintain the health of the host. Altering cellular tropism via high-affinity CD4 binding may be disadvantageous to the virus, disproportionately skewing infection toward a more diseased state.

Herein, as in many other studies, comparative genetics between humans and primates has revealed to us new lessons about HIV’s interaction with its host. This has been a tremendously powerful tool in the HIV field, having led to the original identification of, and mechanistic insights into, many of the host factors that control HIV-1 biology (for only a few examples, see [86,108–112]). As primate research becomes more tenuous [113], we need to keep in mind how powerful these comparative approaches have been in the study of HIV-1 and in the study of other viruses as well (for only a few examples, see [114–117]).

## Materials and methods

### Cells

HEK 293T (ATCC CRL-11268), TZM-bl (NIH ARP # 8129), and canine thymocytes (Cf2Th) (ATCC CRL-1430) were cultured in Dulbecco’s modified Eagle medium (Invitrogen) with 10% FBS, 2 mM L-glutamine, and 1% Pen Strep (complete medium). All cells were maintained at 37°C and 5% CO_2_.

### Isolation of primate CD4+ T cells from whole blood

Whole blood from owl monkey (*Aotus vociferans*) or rhesus macaque (*Macaca mulatta*) donors was received in plastic BD Vacutainer Plus K2EDTA tubes (Cat#366643, lavender cap). The blood samples were obtained from animals housed at the Keeling Center for Comparative Medicine and Research (KCCMR) in Bastrop, TX. Blood was diluted in an equal volume of balanced salt solution (one part solution A [5.5 x 10^−3^ M Anhydrous D-glucose, 5.0 x 10^−5^ M CaCl_2_-2H_2_O, 9.8 x 10^−4^ M MgCl_2_-6H_2_O, 5.4 x 10^−3^ M KCl, 0.145 M TRIS] to nine parts solution B [0.145 M NaCl]). Then, diluted blood was separated in density gradient media (Ficoll-Pacque PLUS GE17-1440-02) by 400 x g centrifugation for 40 minutes. The peripheral blood mononuclear cell layer (i.e., PBMCs or buffy coat) was removed and washed twice in three volumes of balanced salt solution per sample. Each donors’ PBMCs were then immediately subjected to a CD4-positive selection kit (EasySep Human CD4 Positive Selection Kit II Cat #17852) to isolate CD4+ T cells. Rhesus macaque and owl monkey CD4+ T cells were then activated for three days in RPMI supplemented with 100 U IL2/ml (recombinant human-IL2; Sigma #11011456001) and 5 μg/ml or 0.5 μg/ml PHA-P (Sigma L1668), respectively. CD4+ T-cell cultures were subsequently maintained in RPMI + 100 U IL2/ml media.

### Detection of CD4 receptor expression on T cells by flow cytometry

CD4+ T cells isolated from rhesus macaque or owl monkey whole blood (as described above) or human T cells (Hut78 cells) were analyzed for CD4 expression by flow cytometry (Beckman Coulter CyAn ADP High-Performance Flow Cytometer). Cells were first incubated in Fc receptor (Cd16 monoclonal antibody (3G8), eBioscience Cat #16-0166-82) blocking buffer (PBS + 2% FCS + 1mM EDTA + 0.5% BSA) on ice for 1 hour. These cells were then fixed in 1% PFA and stained for cell surface expression of CD4 (BD CD4 mouse anti-human, APC, clone: L200). On the cytometer, only live cells were gated and histograms of CD4 expression determined by unstained and isotype (BD Mouse IgG_1_, Κ, Cat #555751) controls.

### Envelope clones and mutagenesis

The following Env clones were obtained from the NIH AIDS Reagent Program, Division of AIDS, NIAID, NIH: Env clones from early infection (QF495.23M.Env.A1 #11890 and QB857.23I.Env.B3 #11915 [52,118] [Dr. Julie Overbaugh], AC10.0.29 #11024 [53] [Drs. David Montefiori and Dr. Feng Gao], CAP210.2.00.E8 #11317 [36] [Drs. L. Morris, K. Mlisana, and D. Montefiori]), chronic/lab-adapted Env clones commonly used in SHIVs (SF162 #10463 [64–66] [Drs. L. Stamatatos and C. Cheng-Mayer], BaL.26 #11446 [69] [Dr. Mascola], and 89.6 #12485 [71] [Drs. Kathleen Collins and Ronald Collman]), Env clones from chronic infections #12670 (CNE55, CH119, CNE8, X1632 #12670 [49] [Dr. David Montefiori]), and Env clones from maternal and infant/baby transmission pairs #11674 (M/B535, M/BG505, M/BJ613, M/BL274, M/BK184, M/BJ412, M/BI206 [9] [Dr. Julie Overbaugh]). BG505.W6M.B1 [9] and YU2 [31,68] Envs were gifts from Dr. Julie Overbaugh. Single-genome amplification-derived molecular clones of T/F and 6-month chronic viruses (CH40, CH58, CH77, CH470 [3,14,50,51]) were gifts from Dr. Beatrice Hahn. We amplified the Rev-Env cassettes from these by PCR, TA-cloned them into pCR8 (Clontech), and then used Gateway cloning to move them into a gateway converted pCDNA3.1 (Invitrogen) mammalian expression vector. Further description of these Env clones, and those used in the fusion assay screens, are described in S1 Table. Nucleotide changes producing S375Y/W/H and A281V were introduced into BG505.W6M.B1 by site directed mutagenesis using overlapping PCR primers containing the mutation of interest, following standard methods.

### Receptor expression constructs and generation of stable cell lines

CD4 and/or CCR5 was amplified from RNA isolated from the following sources: Jurkat T cells (human CD4, Genbank MK170450), immortalized B-lymphocytes (rhesus macaque CCR5, Genbank NM_001309402.1), whole blood (rhesus macaque CD4 alleles 1–5, Genbank MF632286-MF632290; owl monkey CD4, Genbank MK205145), and fibroblasts (gorilla CD4, Genbank MK170451). Pig-tailed macaque CD4 and CCR5 were subcloned into the pCR8/GW/TOPO TA plasmid from pBabe retroviral vectors provided by Dr. Julie Overbaugh [30]. Human CCR5 was obtained through the NIH AIDS Reagent Program, Division of AIDS, NIAID, NIH (Human CCR5 expression vector pcCCR5 #3325 from Dr. Nathaniel Landau) and subcloned into the pCR8/GW/TOPO TA plasmid. The remaining constructs were purchased as gene block fragments (IDT), PCR amplified (Phusion HiFi Master Mix; Thermo Fisher), and TA cloned into the pCR8/GW/TOPO TA plasmid (Thermo Fisher). The Genbank sequences used for gene block synthesis are as follows: chimpanzee CD4 (NM_001009043.1) and CCR5 (U89797.1), sooty mangabey CD4 (NM_001319342.1) and CCR5 (XM_012033360.1), gorilla CCR5 (AF177901.1), sabaeus CD4 (XM_007967415.1), gelada CD4 (XM_025401282.1), mandrill CD4 (XM_011982990.1), colobus CD4 (XM_011952099.1), baboon CD4 (XM_003905871.3), black snub-nosed monkey CD4 (XM_017891844.1), white-cheeked gibbon CD4 (XM_004092147.1), squirrel monkey CD4 (AF452617.1), marmoset CD4 (AF452616.1), and orangutan CD4 (XM_024256502.1; incomplete CDS, missing 3′ sequence (15 nucleotides) was substituted with human CD4 residues). Following TA cloning, an LR Clonase II reaction (Invitrogen) was used to shuttle inserts into Gateway-converted pLPCX (CD4) or pLHCX (CCR5) retroviral packaging vectors (Clontech).

To produce retroviruses for transduction, HEK293T cells plated in antibiotic free media (1 x 10^6^ cells/well in a 6-well plate) were transfected with a 2 μg of transfer vector (pLPCX [CD4] or pLHCX [CCR5] retroviral vector), 1 μg pCS2-mGP (MLV gag/pol), and 0.2 μg pC-VSVG (VSV-G envelope) using a 3:1 ratio of TransIT 293 (Mirus) transfection reagent to micrograms of DNA, according to manufacturer’s directions. Forty-eight hours post transfection, retrovirus VLPs were collected, filtered through 0.22-μm cellulose acetate filters, and stored at −80°C in single-use aliquots. Cf2Th cells were plated at 3 x 10^4^ cells/well of a 12-well dish (approximately 15% confluent) and 24 hours later, transduced with 500 μl of retroviral supernatant by spinoculation at 1,200 x g for 75 minutes in the presence of 5 μg/ml polybrene. The following day, the cells were placed in complete medium containing antibiotic (3 μg/ml puromycin for pLPCX or 250 μg/ml hygromycin-B for pLHCX) and cultured until stable outgrowth was noted (>1 week). Stable cell lines were maintained indefinitely in selection media.

### Single-cycle HIV-1 infections

To produce HIV-1 reporter viruses, 13 x 10^6^ HEK293T cells were seeded into a 15-cm dish in antibiotic free media and 24 hours later transfected with 5.3 μg of Q23ΔEnv-GFP and 2.7 μg of Env plasmid. Q23ΔEnv-GFP, encoding an approximately 400-bp deletion in Env, and with GFP inserted into the *nef* gene (therefore destroying the function of *nef*), was obtained from Dr. Julie Overbaugh and described previously [30]. Fourty-eight hours post transfection, the cell supernatant was harvested, concentrated (approximately 100-fold) using Amicon Ultracell 100K filters (Millipore), and stored at −80°C in single use aliquots. Virus titers were determined by FACS analysis for GFP-positive cells, using a range of virus dilutions on Cf2Th cells stably expressing human CD4 and CCR5. For infection assays, Cf2Th cells stably expressing CD4 and CCR5 were plated at 3 x 10^4^ cells/well of a 48-well plate (approximately 60% confluent) 24 hours prior to infection. The cells were then infected with HIV-1 pseudoviruses at a multiplicity of infection (MOI) of approximately 0.6 in the presence of 5 μg/ml of polybrene by spinoculation at 1,200 x g for 75 minutes. Fourty-eight hours post infection, the cells were harvested from the plate and fixed in 2% paraformaldehyde for 10 minutes. The fixed cells were washed three times with PBS and resuspended in 50 μl FACS buffer (1X PBS buffer containing 2% FBS and 1 mM EDTA) antibody cocktail. All antibody pairs were used at 1:200 dilutions, and incubated at 4°C with cells for 30 minutes as follows: for primate CD4/primate CCR5 (S2 Fig), PeCy7 mouse α-human CD4 clone L200 (BD #560644) and APC mouse α-human CD195 (CCR5) clone 3A9 (BD #560748); for all other studies, the combination of APC mouse α-human CD4 clone L200 (BD #551980) and PeCy7 mouse α-human CD195 (CCR5) 2D7 (BD #557752) was used. Stained cells were analyzed on a CyAn ADP (Beckman Coulter) flow cytometer. Following live-cell gating, CD4 and CCR5 expressing gates were drawn and then the percent GFP positive cells was enumerated within the double positive population. The data from approximately 1 x 10^4^ live cells was analyzed using FlowJo version 10.

### Genotype and allele determination of Indian-origin macaque CD4

PAXgene (BD #762165) preserved whole blood was obtained from animals housed at the KCCMR per blood collection protocols approved by the University of Texas, MD Anderson Cancer Center IACUC. Total DNA/RNA was extracted using the AllPrep DNA/RNA mini kit (Qiagen). Extracted RNA was used as a template for oligo (dt) primed reverse transcription (SuperScript III RT; Thermo Fisher). PCR amplification of CD4 was performed using PCR Supermix HiFi (Thermo Fisher) containing 1–10 ng of cDNA and 0.2 μM final concentration of each PCR primer CD4-Forward 5′ AAGCAGCGGGCAAGAAAGACG 3′ and CD4-Reverse 5′ CAAGTTCCTGCCCTCTGTGG 3′ in a final volume of 25 μl. PCR cleanup was performed using Exonuclease-I and Shrimp Alkaline Phosphatase treatment (Affymetrix) for 15 minutes at 37°C, and then the cleaned-up products were Sanger sequenced using the following CD4-Forward 5′ GGAGTTCAAAATAGACATCG 3′ and CD4-Reverse 5′ CAGACACTTCCTTGTTCTTC 3′ sequencing primers. The resulting genotype information was used to phase SNPs using Phase 2.1 [81,82] in DNAsp v5 [83]. Five unique rhesus macaque CD4 alleles were identified, TA cloned into the pCR8 gateway Topo TA cloning vector (Thermo Fisher), and then subcloned into the gateway converted pLPCX retroviral vector (Clontech). All constructs were further verified by Sanger sequencing. Allele 1 represented the major allele circulating in this population and was used in all experiments except where designated otherwise.

### Production of sCD4 and virus neutralization assays

Human, chimpanzee, and rhesus macaque CD4 cloned into the pCR8 plasmid (described above) served as a template for soluble CD4 (sCD4) PCR. The D1/D2 domain (nucleotide positions 75–603, minus signal peptide) was amplified by PCR and cloned into pHL-sec [119] (Addgene #99845), which has been optimized for protein production in mammalian cells. 13 x 10^6^ 293T cells (three 15-cm dishes) were transfected with 25 μg of sCD4 expression plasmids using TransIT-293 (Mirus), and cell supernatant containing secreted sCD4 was harvested at days three and six post transfection. Cell supernatant was spun down at 1,200 x g for 5 minutes to remove cell debris, filtered using a 0.45-μm cellulose acetate membrane, and mixed 1:1 with wash buffer (25 mM Na_3_PO_4_ pH 7.4, 500 mM NaCl, 20 mM imidazole). Cleared cell supernatant was then incubated with 1-ml bed volume of Ni-NTA agarose beads (Qiagen, #30210) equilibrated in wash buffer for 2 hours at 4°C. The mixture was then added to a gravity flow chromatography column and the flow-through was passed through the column a second time. The Ni-NTA beads were washed with 50 ml of wash buffer. Bound protein was eluted with wash buffer containing 250 mM imidazole in 1-ml fractions. Fractions containing sCD4 were pooled, washed three times with PBS, and concentrated to 500 μl using an Amicon Ultra-15 ml centrifugal filter with a 10 kDa molecular weight cutoff (EMD Millipore, #UFC901008). The sample was purified to homogeneity on a Superdex 75 Increase 10/300 GL (GE Healthcare, #29-1487-21) in PBS. Purified samples were concentrated to 1 mg/ml using an Amicon Ultra 0.5-ml centrifugal filter with a 10-kDa molecular weight cutoff, aliquoted for single use, and flash-frozen in liquid nitrogen.

For the neutralization assays, each virus (normalized by equivalent TDU/ml) was incubated with serial 2-fold dilutions of sCD4 (range 30 μM to approximately 1 μM final concentration) at 37°C for 1 hour. Human, chimpanzee, and rhesus sCD4 were produced in this study (methods above), and commercially available human sCD4 was obtained through the National Institutes of Health AIDS Reagent Program: sCD4-183 #7356 (from Pharmacia, Inc.). Following incubation, the sCD4 treated and untreated viruses were spinoculated (1,200 x g for 75 minutes) onto 1 x 10^4^ TZM-bl cells plated in 96-well plates, in the presence of 5 μg/ml of polybrene. Following spinoculation, the cells were washed three times with PBS and given fresh media. At 48 hours post infection, infectivity was measured by firefly luciferase assays following the manufacturers protocol (Promega). Luminescence was determined using the BMG Clariostar plate reader.

### Cell-cell fusion assay

Cf2Th cells stably expressing human or primate CD4 and human CCR5, and 293T cells, were plated at 3 x 10^5^ and 1 x 10^6^ cells/well of a 6-well dish, respectively. The next day, 1) Cf2Th cells were transfected with 2.5 μg of DNA (1.25 μg of ½ renilla luciferase [79] and 1.25 μg of pCDNA3.1 filler DNA) using Lipofectamine 3000 (Invitrogen) and 2) 293T cells were transfected with 2.5 μg of DNA (1.25 μg of ½ renilla luciferase and 1.25 μg of the Env expression plasmid) using TransIT 293 (Mirus). Twenty-four hours post transfection, the cells were removed from the plate using an enzyme free 1X citric saline solution (10X solution, 1.35M KCl, 0.15M Sodium Citrate) diluted in PBS, counted, and resuspended to a final volume of 1x10^5^ cells/ml. 100 μl of transfected cells in quadrupilicate (1 x 10^4^ 293T and Cf2Th cells) were mixed 1:1 and incubated for 6 hours in 96-well flat bottom plates. Following incubation, fusion was assessed using a Renilla luciferase assay (Promega). Luminescence was determined using the BMG Clariostar plate reader.

### Data presentation and analysis

All data were plotted, and one-way ANOVA and Mann–Whitney U tests were performed where indicated, using Prism version 7.0a for mac (GraphPad). IC_50_ values were determined by first normalizing the relative luminescence units as follows: 100% and 0% inhibition was defined by wells receiving the highest concentration of sCD4 and absence of sCD4, respectively. A nonlinear regression curve was fit to the normalized data, and IC_50_ values were calculated using Prism (GraphPad) software.

### Ethics statement

The blood collections performed on the animals in this study were approved as protocol number 00000451-RN02 by the University of Texas, MD Anderson Cancer Center, Institutional Animal Care and Use Committee (MDACC-IACUC). The protocol approved by the MDACC-IACUC adhered to the recommendations provided in the *Guide for the Care and Use of Laboratory Animals* [120] and also adhered to the blood collection volumes recommended by the Guidance Document of the Association of Primate Veterinarians (https://www.primatevets.org/guidance-documents) entitled *Guidelines for Blood Sampling in Nonhuman Primates* (also known as *Blood Sampling Guidelines for Nonhuman Primates in Biomedical Research*).

## Supporting information

S1 Fig Stable expression of human and primate CD4 receptors. (A) Histograms of CD4 expression levels in Cf2Th cell lines engineered to stably express each primate CD4 receptor with human CCR5. (B) Gating strategy for enumerating the GFP+ (infected) cell population. Single stains of human CD4 (blue) and human CCR5 (red) as well as unstained cells (black) were used to denote receptor expression quadrants. A CD4 window was drawn such that it would capture equivalent CD4/CCR5 receptor expression across all stable cell lines shown in (A). GFP+ cells were enumerated within this window. (C) Surface expression of CD4 from human T cells (Hut-78 cells) and primate T cells that we isolated directly from primate blood. (D) Histograms of CD4 expression levels in Cf2Th cell lines with (red line) and without (black line) HIV-1 infection. Shaded histograms are from cells transduced with an empty vector control and denote the CD4-negative population. Data associated with this figure can be found in the supplemental data file (S7 Data). CCR5, C-C motif chemokine receptor 5; GFP, green fluorescent protein.
(TIF)

S2 Fig Stable expression of primate CD4 and CCR5 receptors. (A) Histograms of CD4 and CCR5 expression levels in Cf2Th cell lines made to stably express primate CD4/CCR5 receptor pairs from each primate species (top) or each primate CD4 paired with human CCR5 (bottom). (B) Cf2Th cell lines stably expressing primate CD4/CCR5 receptor pairs from each primate species (black bars) and each primate CD4 paired with human CCR5 (gray bars) were infected with HIV-1 GFP pseudotyped with a subtype A Envelope (BG505). Error bars represent the mean + SEM from two independent experiments, each with three technical replicates. Data associated with this figure can be found in the supplemental data file (S8 and S9 Datas). CCR5, C-C motif chemokine receptor 5; GFP, green fluorescent protein.
(TIF)

S3 Fig Production of human and primate sCD4 receptors. (A, B, C) Size-exclusion profiles of (A) human, (B) chimpanzee, and (C) rhesus macaque soluble CD4 proteins (sCD4). The input sample is made from combined fractions eluted from the Ni-NTA column. Total protein was visualized using the TGX stain-free system (Bio-Rad). Fractions collected from the Superdex 75 column are each 1 ml. Fractions indicated with a red box eluted at a volume consistent with the molecular weight of sCD4 monomers and were combined for use in downstream experiments. Plots below the gels are A280 absorbance readings from the FPLC spectrophotometer. (D) Total protein stain of purified sCD4 molecules. Human, chimpanzee, and rhesus macaque CD4 are all differentially glycosylated, explaining the differences in migration [48]. (E) HIV-1 pseudotyped with the indicated Envs (top of graphs), was preincubated with increasing concentrations of human sCD4 produced in this study (solid line; see panel A) or a commercially available sCD4 obtained from National Institutes of Health AIDS Reagent Program (#7356) (dashed line), and then used to infect TZM-bl cells. Error bars represent the SD from *n* = 4 technical replicates. Data associated with this figure can be found in the supplemental data file (S10 Data). FPLC, fast protein liquid chromatography; TGX, Tris-Glycine eXtended
(TIF)

S1 Table Envelope clones used in this study. (DOCX)

S1 Data Raw values for the data in Fig 1. (PZFX)

S2 Data Raw values for the data in Fig 2. (PZFX)

S3 Data Raw values for the data in Fig 3. (PZFX)

S4 Data Raw values for the data in Fig 4. (PZFX)

S5 Data Raw values for the data in Fig 5. (PZFX)

S6 Data Raw values for the data in Fig 6. (PZFX)

S7 Data Flow cytometry files corresponding to S1 Fig. (ZIP)

S8 Data Flow cytometry files corresponding to S2 Fig. (ZIP)

S9 Data Raw values for the data in S2 Fig. (PZFX)

S10 Data Raw values for the data in S3 Fig. (PZFX)

## Abbreviations

CCR5: C-C motif chemokine receptor 5
CXCR4: C-X-C motif chemokine receptor 4
Env: Envelope
GFP: green fluorescent protein
PBMC: peripheral blood mononuclear cells
sCD4: soluble CD4
SIV: simian immunodeficiency virus

## References

[1] Sagar M. HIV-1 transmission biology: selection and characteristics of infecting viruses. J Infect Dis. 2010; 202 Suppl 2: S289–96. 20846035 10.1086/655656 PMC2946383

[2] Kearney M, Maldarelli F, Shao W, Margolick JB, Daar ES, Mellors JW, et al Human Immunodeficiency Virus Type 1 Population Genetics and Adaptation in Newly Infected Individuals. J Virol. 2009; 83: 2715–2727. 10.1128/JVI.01960-08 19116249 PMC2648286

[3] Keele BF, Giorgi EE, Salazar-Gonzalez JF, Decker JM, Pham KT, Salazar MG, et al Identification and characterization of transmitted and early founder virus envelopes in primary HIV-1 infection. Proc Natl Acad Sci USA. 2008; 105: 7552–7557. 10.1073/pnas.0802203105 18490657 PMC2387184

[4] Abrahams MR, Anderson JA, Giorgi EE, Seoighe C, Mlisana K, Ping LH, et al Quantitating the multiplicity of infection with human immunodeficiency virus type 1 subtype C reveals a non-poisson distribution of transmitted variants. J Virol. 2009; 83: 3556–3567. 10.1128/JVI.02132-08 19193811 PMC2663249

[5] Derdeyn CA, Decker JM, Bibollet-Ruche F, Mokili JL, Muldoon M, Denham SA, et al Envelope-constrained neutralization-sensitive HIV-1 after heterosexual transmission. Science. 2004; 303: 2019–2022. 10.1126/science.1093137 15044802

[6] Tully DC, Ogilvie CB, Batorsky RE, Bean DJ, Power KA, Ghebremichael M, et al Differences in the Selection Bottleneck between Modes of Sexual Transmission Influence the Genetic Composition of the HIV-1 Founder Virus. PLoS Pathog. 2016; 12: e1005619 10.1371/journal.ppat.1005619 27163788 PMC4862634

[7] Bar KJ, Li H, Chamberland A, Tremblay C, Routy JP, Grayson T, et al Wide variation in the multiplicity of HIV-1 infection among injection drug users. J Virol. 2010; 84: 6241–6247. 10.1128/JVI.00077-10 20375173 PMC2876625

[8] Masharsky AE, Dukhovlinova EN, Verevochkin SV, Toussova OV, Skochilov RV, Anderson JA, et al A Substantial Transmission Bottleneck among Newly and Recently HIV-1-Infected Injection Drug Users in St Petersburg, Russia. J Infect Dis. 2010; 201: 1697–1702. 10.1086/652702 20423223

[9] Wu X, Parast AB, Richardson BA, Nduati R, John-Stewart G, Mbori-Ngacha D, et al Neutralization escape variants of human immunodeficiency virus type 1 are transmitted from mother to infant. J Virol. 2006; 80: 835–844. 10.1128/JVI.80.2.835-844.2006 16378985 PMC1346878

[10] Parker ZF, Iyer SS, Wilen CB, Parrish NF, Chikere KC, Lee FH, et al Transmitted/Founder and Chronic HIV-1 Envelope Proteins Are Distinguished by Differential Utilization of CCR5. J Virol. 2013; 87: 2401–2411. 10.1128/JVI.02964-12 23269796 PMC3571396

[11] Go EP, Hewawasam G, Liao H-X, Chen H, Ping L-H, Anderson JA, et al Characterization of glycosylation profiles of HIV-1 transmitted/founder envelopes by mass spectrometry. J Virol. 2011; 85: 8270–8284. 10.1128/JVI.05053-11 21653661 PMC3147976

[12] Liu Y, Curlin ME, Diem K, Zhao H, Ghosh AK, Zhu H, et al Env length and N-linked glycosylation following transmission of human immunodeficiency virus Type 1 subtype B viruses. Virology. 2008; 374: 229–233. 10.1016/j.virol.2008.01.029 18314154 PMC2441482

[13] Liao HX, Tsao CY, Alam SM, Muldoon M, Vandergrift N, Ma BJ, et al Antigenicity and Immunogenicity of Transmitted/Founder, Consensus, and Chronic Envelope Glycoproteins of Human Immunodeficiency Virus Type 1. J Virol. 2013; 87: 4185–4201. 10.1128/JVI.02297-12 23365441 PMC3624376

[14] Foster TL, Wilson H, Iyer SS, Coss K, Doores K, Smith S, et al Resistance of Transmitted Founder HIV-1 to IFITM-Mediated Restriction. Cell Host Microbe. 2016; 20: 429–442. 10.1016/j.chom.2016.08.006 27640936 PMC5075283

[15] Fenton-May AE, Dibben O, Emmerich T, Ding H, Pfafferott K, Aasa-Chapman MM, et al Relative resistance of HIV-1 founder viruses to control by interferon-alpha. Retrovirology. 2013; 10: 146 10.1186/1742-4690-10-146 24299076 PMC3907080

[16] Warren CJ, Sawyer SL. How host genetics dictates successful viral zoonosis. PLoS Biol. 2019; 17: e3000217 10.1371/journal.pbio.3000217 31002666 PMC6474636

[17] Sharp PM, Hahn BH. Origins of HIV and the AIDS pandemic. Cold Spring Harb Perspect Med. 2011; 1: a006841 10.1101/cshperspect.a006841 22229120 PMC3234451

[18] Hatziioannou T, Evans DT. Animal models for HIV/AIDS research. Nat Rev Micro. 2012; 10: 852–867. 10.1038/nrmicro2911 23154262 PMC4334372

[19] Bell SM, Bedford T. Modern-day SIV viral diversity generated by extensive recombination and cross-species transmission. PLoS Pathog. 2017;13: e1006466 10.1371/journal.ppat.1006466 28672035 PMC5510905

[20] Zhang ZD, Weinstock G, Gerstein M. Rapid evolution by positive Darwinian selection in T-cell antigen CD4 in primates. J Mol Evol. 2008; 66: 446–456. 10.1007/s00239-008-9097-1 18414925

[21] Meyerson NR, Rowley PA, Swan CH, Le DT, Wilkerson GK, Sawyer SL. Positive selection of primate genes that promote HIV-1 replication. Virology. 2014; 454–455: 291–298. 10.1016/j.virol.2014.02.029 24725956 PMC4028154

[22] Demogines A, Abraham J, Choe H, Farzan M, Sawyer SL. Dual host-virus arms races shape an essential housekeeping protein. PLoS Biol. 2013; 11: e1001571 10.1371/journal.pbio.1001571 23723737 PMC3665890

[23] Demogines A, Farzan M, Sawyer SL. Evidence for ACE2-utilizing coronaviruses (CoVs) related to severe acute respiratory syndrome CoV in bats. J Virol. 2012; 86: 6350–6353. 10.1128/JVI.00311-12 22438550 PMC3372174

[24] Kaelber JT, Demogines A, Harbison CE, Allison AB, Goodman LB, Ortega AN, et al Evolutionary reconstructions of the transferrin receptor of Caniforms supports canine parvovirus being a re-emerged and not a novel pathogen in dogs. PLoS Pathog. 2012; 8: e1002666 10.1371/journal.ppat.1002666 22570610 PMC3342950

[25] Kerr SA, Jackson EL, Lungu OI, Meyer AG, Demogines A, Ellington AD, et al Computational and Functional Analysis of the Virus-Receptor Interface Reveals Host Range Trade-Offs in New World Arenaviruses. J Virol. 2015; 89: 11643–11653. 10.1128/JVI.01408-15 26355089 PMC4645654

[26] Martin C, Buckler-White A, Wollenberg K, Kozak CA. The avian XPR1 gammaretrovirus receptor is under positive selection and is disabled in bird species in contact with virus-infected wild mice. J Virol. 2013; 87: 10094–10104. 10.1128/JVI.01327-13 23843647 PMC3754004

[27] Meyerson NR, Sawyer SL. Two-stepping through time: mammals and viruses. Trends Microbiol. 2011; 19: 286–294. 10.1016/j.tim.2011.03.006 21531564 PMC3567447

[28] Liu Q, Acharya P, Dolan MA, Zhang P, Guzzo C, Lu J, et al Quaternary contact in the initial interaction of CD4 with the HIV-1 envelope trimer. Nat Struct Mol Biol. 2017; 24: 370–378. 10.1038/nsmb.3382 28218750 PMC5798227

[29] Joseph SB, Swanstrom R. The evolution of HIV-1 entry phenotypes as a guide to changing target cells. J Leukoc Biol. 2018; 103: 421–431. 10.1002/JLB.2RI0517-200R 29389021

[30] Humes D, Overbaugh J. Adaptation of Subtype A Human Immunodeficiency Virus Type 1 Envelope to Pig-Tailed Macaque Cells. J Virol. 2011; 85: 4409–4420. 10.1128/JVI.02244-10 21325401 PMC3126259

[31] Humes D, Emery S, Laws E, Overbaugh J. A Species-Specific Amino Acid Difference in the Macaque CD4 Receptor Restricts Replication by Global Circulating HIV-1 Variants Representing Viruses from Recent Infection. J Virol. 2012; 86: 12472–12483. 10.1128/JVI.02176-12 22973036 PMC3497638

[32] Del Prete GQ, Ailers B, Moldt B, Keele BF, Estes JD, Rodriguez A, et al Selection of Unadapted, Pathogenic SHIVs Encoding Newly Transmitted HIV-1 Envelope Proteins. Cell Host Microbe. 2014; 16: 412–418. 10.1016/j.chom.2014.08.003 25211081 PMC4268878

[33] Meyerson NR, Sharma A, Wilkerson GK, Overbaugh J, Sawyer SL. Identification of Owl Monkey CD4 Receptors Broadly Compatible with Early-Stage HIV-1 Isolates. J Virol. 2015; 89: 8611–8622. 10.1128/JVI.00890-15 26063421 PMC4524261

[34] Cohen MS, Gay CL, Busch MP, Hecht FM. The detection of acute HIV infection. J Infect Dis. 2010; 202 Suppl 2: S270–7. 10.1086/655651 20846033

[35] Fiebig EW, Wright DJ, Rawal BD, Garrett PE, Schumacher RT, Peddada L, et al Dynamics of HIV viremia and antibody seroconversion in plasma donors: implications for diagnosis and staging of primary HIV infection. AIDS. 2003; 17: 1871–1879. 10.1097/01.aids.0000076308.76477.b8 12960819

[36] Li M, Salazar-Gonzalez JF, Derdeyn CA, Morris L, Williamson C, Robinson JE, et al Genetic and neutralization properties of subtype C human immunodeficiency virus type 1 molecular env clones from acute and early heterosexually acquired infections in Southern Africa. J Virol. 2006;80: 11776–11790. 10.1128/JVI.01730-06 16971434 PMC1642599

[37] Etemad B, Fellows A, Kwambana B, Kamat A, Feng Y, Lee S, et al Human immunodeficiency virus type 1 V1-to-V5 envelope variants from the chronic phase of infection use CCR5 and fuse more efficiently than those from early after infection. J Virol. 2009; 83: 9694–9708. 10.1128/JVI.00925-09 19625411 PMC2748008

[38] Wang H, Cohen AA, Galimidi RP, Gristick HB, Jensen GJ, Bjorkman PJ. Cryo-EM structure of a CD4-bound open HIV-1 envelope trimer reveals structural rearrangements of the gp120 V1V2 loop. Proc Natl Acad Sci USA. 2016; 113: E7151–E7158. 10.1073/pnas.1615939113 27799557 PMC5135367

[39] Sanders RW, Derking R, Cupo A, Julien J-P, Yasmeen A, de Val N, et al A next-generation cleaved, soluble HIV-1 Env trimer, BG505 SOSIP.664 gp140, expresses multiple epitopes for broadly neutralizing but not non-neutralizing antibodies. PLoS Pathog. 2013; 9: e1003618 10.1371/journal.ppat.1003618 24068931 PMC3777863

[40] Sanders RW, Moore JP. Native-like Env trimers as a platform for HIV-1 vaccine design. Immunol Rev. 2017; 275: 161–182. 10.1111/imr.12481 28133806 PMC5299501

[41] Lu M, Ma X, Castillo-Menendez LR, Gorman J, Alsahafi N, Ermel U, et al Associating HIV-1 envelope glycoprotein structures with states on the virus observed by smFRET. Nature. 2019; 568: 415–419. 10.1038/s41586-019-1101-y 30971821 PMC6655592

[42] Poss M, Overbaugh J. Variants from the diverse virus population identified at seroconversion of a clade A human immunodeficiency virus type 1-infected woman have distinct biological properties. J Virol. 1999; 73: 5255–5264. 10364271 10.1128/jvi.73.7.5255-5264.1999 PMC112580

[43] Ryu SE, Kwong PD, Truneh A, Porter TG, Arthos J, Rosenberg M, et al Crystal structure of an HIV-binding recombinant fragment of human CD4. Nature. 1990; 348: 419–426. 10.1038/348419a0 2247146 PMC5638305

[44] Wang JH, Yan YW, Garrett TP, Liu JH, Rodgers DW, Garlick RL, et al Atomic structure of a fragment of human CD4 containing two immunoglobulin-like domains. Nature. 1990; 348: 411–418. 10.1038/348411a0 1701030

[45] Kwong PD, Wyatt R, Robinson J, Sweet RW, Sodroski J, Hendrickson WA. Structure of an HIV gp120 envelope glycoprotein in complex with the CD4 receptor and a neutralizing human antibody. Nature. 1998; 393: 648–659. 10.1038/31405 9641677 PMC5629912

[46] Arthos J, Deen KC, Chaikin MA, Fornwald JA, Sathe G, Sattentau QJ, et al Identification of the residues in human CD4 critical for the binding of HIV. Cell. 1989; 57: 469–481. 2541915 10.1016/0092-8674(89)90922-7

[47] Landau NR, Warton M, Littman DR. The envelope glycoprotein of the human immunodeficiency virus binds to the immunoglobulin-like domain of CD4. Nature. 1988; 334: 159–162. 10.1038/334159a0 3260352

[48] Warren CJ, Meyerson NR, Stabell AC, Fattor WT, Wilkerson GK, Sawyer SL. A glycan shield on chimpanzee CD4 protects against infection by primate lentiviruses (HIV/SIV). Proc Natl Acad Sci USA. 2019; 116: 3229–3238. 10.1073/pnas.1821197116 31113887 PMC6561292

[49] deCamp A, Hraber P, Bailer RT, Seaman MS, Ochsenbauer C, Kappes J, et al Global panel of HIV-1 Env reference strains for standardized assessments of vaccine-elicited neutralizing antibodies. J Virol. 2014;88: 2489–2507. 10.1128/JVI.02853-13 24352443 PMC3958090

[50] Parrish NF, Gao F, Li H, Giorgi EE, Barbian HJ, Parrish EH, et al Phenotypic properties of transmitted founder HIV-1. Proc Natl Acad Sci USA. 2013;110: 6626–6633. 10.1073/pnas.1304288110 23542380 PMC3637789

[51] Freel SA, Picking RA, Ferrari G, Ding H, Ochsenbauer C, Kappes JC, et al Initial HIV-1 antigen-specific CD8+ T cells in acute HIV-1 infection inhibit transmitted/founder virus replication. J Virol. 2012; 86: 6835–6846. 10.1128/JVI.00437-12 22514337 PMC3393529

[52] Blish CA, Jalalian-Lechak Z, Rainwater S, Nguyen M-A, Dogan OC, Overbaugh J. Cross-subtype neutralization sensitivity despite monoclonal antibody resistance among early subtype A, C, and D envelope variants of human immunodeficiency virus type 1. J Virol. 2009; 83: 7783–7788. 10.1128/JVI.00673-09 19474105 PMC2708608

[53] Li M, Gao F, Mascola JR, Stamatatos L, Polonis VR, Koutsoukos M, et al Human immunodeficiency virus type 1 env clones from acute and early subtype B infections for standardized assessments of vaccine-elicited neutralizing antibodies. J Virol. 2005; 79: 10108–10125. 10.1128/JVI.79.16.10108-10125.2005 16051804 PMC1182643

[54] Swanstrom R, Graham WD, Zhou S. Sequencing the Biology of Entry: The Retroviral env Gene. Curr Top Microbiol Immunol. 2017; 407: 65–82. 10.1007/82_2017_35 28688086 PMC7122457

[55] Ping L-H, Joseph SB, Anderson JA, Abrahams M-R, Salazar-Gonzalez JF, Kincer LP, et al Comparison of viral Env proteins from acute and chronic infections with subtype C human immunodeficiency virus type 1 identifies differences in glycosylation and CCR5 utilization and suggests a new strategy for immunogen design. J Virol. 2013; 87: 7218–7233. 10.1128/JVI.03577-12 23616655 PMC3700278

[56] Parrish NF, Wilen CB, Banks LB, Iyer SS, Pfaff JM, Salazar-Gonzalez JF, et al Transmitted/founder and chronic subtype C HIV-1 use CD4 and CCR5 receptors with equal efficiency and are not inhibited by blocking the integrin α4β7. PLoS Pathog. 2012; 8: e1002686 10.1371/journal.ppat.1002686 22693444 PMC3364951

[57] Sheppard HW, Celum C, Michael NL, O'Brien S, Dean M, Carrington M, et al HIV-1 infection in individuals with the CCR5-Delta32/Delta32 genotype: acquisition of syncytium-inducing virus at seroconversion. J AIDS. 2002; 29: 307–313. 10.1097/00126334-200203010-00013 11873082

[58] Oh D-Y, Jessen H, Kücherer C, Neumann K, Oh N, Poggensee G, et al CCR5Delta32 genotypes in a German HIV-1 seroconverter cohort and report of HIV-1 infection in a CCR5Delta32 homozygous individual. PLoS ONE. 2008; 3: e2747 10.1371/journal.pone.0002747 18648518 PMC2453227

[59] Huang W, Eshleman SH, Toma J, Stawiski E, Whitcomb JM, Jackson JB, et al Vertical transmission of X4-tropic and dual-tropic HIV-1 in five Ugandan mother-infant pairs. AIDS. 2009; 23: 1903–1908. 10.1097/QAD.0b013e32832f1802 19593079 PMC2764460

[60] Huang W, Toma J, Stawiski E, Fransen S, Wrin T, Parkin N, et al Characterization of human immunodeficiency virus type 1 populations containing CXCR4-using variants from recently infected individuals. AIDS Res Hum Retroviruses. 2009; 25: 795–802. 10.1089/aid.2008.0252 19678765 PMC2827835

[61] de Mendoza C, Rodriguez C, García F, Eiros JM, Ruiz L, Caballero E, et al Prevalence of X4 tropic viruses in patients recently infected with HIV-1 and lack of association with transmission of drug resistance. J Antimicrob Chemother. 2007; 59: 698–704. 10.1093/jac/dkm012 17327295

[62] Satomi M, Shimizu M, Shinya E, Watari E, Owaki A, Hidaka C, et al Transmission of macrophage-tropic HIV-1 by breast-milk macrophages via DC-SIGN. J Infect Dis. 2005; 191: 174–181. 10.1086/426829 15609226

[63] Peters PJ, Sullivan WM, Dueñas-Decamp MJ, Bhattacharya J, Ankghuambom C, Brown R, et al Non-macrophage-tropic human immunodeficiency virus type 1 R5 envelopes predominate in blood, lymph nodes, and semen: implications for transmission and pathogenesis. J Virol. 2006; 80: 6324–32. 10.1128/JVI.02328-05 16775320 PMC1488974

[64] Cheng-Mayer C, Liu R, Landau NR, Stamatatos L. Macrophage tropism of human immunodeficiency virus type 1 and utilization of the CC-CKR5 coreceptor. J Virol. 1997; 71: 1657–1661. 8995695 10.1128/jvi.71.2.1657-1661.1997 PMC191226

[65] Stamatatos L, Lim M, Cheng-Mayer C. Generation and structural analysis of soluble oligomeric gp140 envelope proteins derived from neutralization-resistant and neutralization-susceptible primary HIV type 1 isolates. AIDS Res Hum Retroviruses. 2000; 16: 981–994. 10.1089/08892220050058407 10890360

[66] Stamatatos L, Wiskerchen M, Cheng-Mayer C. Effect of major deletions in the V1 and V2 loops of a macrophage-tropic HIV type 1 isolate on viral envelope structure, cell entry, and replication. AIDS Res Hum Retroviruses. 1998; 14: 1129–1139. 10.1089/aid.1998.14.1129 9737584

[67] Cheng-Mayer C, Levy JA. Distinct biological and serological properties of human immunodeficiency viruses from the brain. Ann Neurol. 1988; 23: S58–S61. 3258140 10.1002/ana.410230716

[68] Li Y, Hui H, Burgess CJ, Price RW, Sharp PM, Hahn BH, et al Complete nucleotide sequence, genome organization, and biological properties of human immunodeficiency virus type 1 in vivo: evidence for limited defectiveness and complementation. J Virol. 1992; 66: 6587–6600. 1404605 10.1128/jvi.66.11.6587-6600.1992 PMC240154

[69] Li Y, Svehla K, Mathy NL, Voss G, Mascola JR, Wyatt R. Characterization of antibody responses elicited by human immunodeficiency virus type 1 primary isolate trimeric and monomeric envelope glycoproteins in selected adjuvants. J Virol. 2006; 80: 1414–1426. 10.1128/JVI.80.3.1414-1426.2006 16415019 PMC1346938

[70] Gartner S, Markovits P, Markovitz DM, Kaplan MH, Gallo RC, Popovic M. The role of mononuclear phagocytes in HTLV-III/LAV infection. Science. 1986; 233: 215–219. 3014648 10.1126/science.3014648

[71] Carter CC, Onafuwa-Nuga A, McNamara LA, Riddell J, Bixby D, Savona MR, et al HIV-1 infects multipotent progenitor cells causing cell death and establishing latent cellular reservoirs. Nat Med. 2010; 16: 446–451. 10.1038/nm.2109 20208541 PMC2892382

[72] Collman R, Balliet JW, Gregory SA, Friedman H, Kolson DL, Nathanson N, et al An infectious molecular clone of an unusual macrophage-tropic and highly cytopathic strain of human immunodeficiency virus type 1. J Virol. 1992; 66: 7517–7521. 1433527 10.1128/jvi.66.12.7517-7521.1992 PMC240461

[73] Salimi H, Roche M, Webb N, Gray LR, Chikere K, Sterjovski J, et al Macrophage-tropic HIV-1 variants from brain demonstrate alterations in the way gp120 engages both CD4 and CCR5. J Leukoc Biol. 2013; 93: 113–126. 10.1189/jlb.0612308 23077246 PMC3525831

[74] Joseph SB, Arrildt KT, Swanstrom AE, Schnell G, Lee B, Hoxie JA, et al Quantification of entry phenotypes of macrophage-tropic HIV-1 across a wide range of CD4 densities. J Virol. 2014; 88: 1858–1869. 10.1128/JVI.02477-13 24307580 PMC3911544

[75] Thomas ER, Dunfee RL, Stanton J, Bogdan D, Taylor J, Kunstman K, et al Macrophage entry mediated by HIV Envs from brain and lymphoid tissues is determined by the capacity to use low CD4 levels and overall efficiency of fusion. Virology. 2007; 360: 105–119. 10.1016/j.virol.2006.09.036 17084877 PMC1890014

[76] Smith DH, Byrn RA, Marsters SA, Gregory T, Groopman JE, Capon DJ. Blocking of HIV-1 infectivity by a soluble, secreted form of the CD4 antigen. Science. 1987; 238: 1704–1707. 3500514 10.1126/science.3500514

[77] Li H, Wang S, Kong R, Ding W, Lee F-H, Parker Z, et al Envelope residue 375 substitutions in simian-human immunodeficiency viruses enhance CD4 binding and replication in rhesus macaques. Proc Natl Acad Sci USA. 2016; 113: E3413–22. 10.1073/pnas.1606636113 27247400 PMC4914158

[78] Del Prete GQ, Keele BF, Fode J, Thummar K, Swanstrom AE, Rodriguez A, et al A single gp120 residue can affect HIV-1 tropism in macaques. PLoS Pathog. 2017; 13: e1006572 10.1371/journal.ppat.1006572 28945790 PMC5629034

[79] Kondo N, Miyauchi K, Meng F, Iwamoto A, Matsuda Z. Conformational changes of the HIV-1 envelope protein during membrane fusion are inhibited by the replacement of its membrane-spanning domain. J Biol Chem. 2010; 285: 14681–14688. 10.1074/jbc.M109.067090 20197275 PMC2863243

[80] Bibollet-Ruche F, Russell RM, Liu W, Stewart-Jones GBE, Sherrill-Mix S, Li Y, et al CD4 receptor diversity in chimpanzees protects against SIV infection. Proc Natl Acad Sci USA. 2019; 11: 201821197 10.1073/pnas.1821197116 30718403 PMC6386711

[81] Stephens M, Smith NJ, Donnelly P. A new statistical method for haplotype reconstruction from population data. Am J Hum Genet. 2001; 68: 978–989. 10.1086/319501 11254454 PMC1275651

[82] Stephens M, Donnelly P. A comparison of bayesian methods for haplotype reconstruction from population genotype data. Am J Hum Genet. 2003; 73: 1162–1169. 10.1086/379378 14574645 PMC1180495

[83] Librado P, Rozas J. DnaSP v5: a software for comprehensive analysis of DNA polymorphism data. Bioinformatics. 2009; 25: 1451–1452. 10.1093/bioinformatics/btp187 19346325

[84] Malim MH, Bieniasz PD. HIV Restriction Factors and Mechanisms of Evasion. Cold Spring Harb Perspect Med. 2012; 2: a006940–a006940. 10.1101/cshperspect.a006940 22553496 PMC3331687

[85] Saito A, Akari H. Macaque-tropic human immunodeficiency virus type 1: breaking out of the host restriction factors. Front Microbiol. Frontiers; 2013; 4: 187 10.3389/fmicb.2013.00187 23847610 PMC3705164

[86] Sawyer SL, Wu LI, Emerman M, Malik HS. Positive selection of primate TRIM5alpha identifies a critical species-specific retroviral restriction domain. Proc Natl Acad Sci USA. 2005; 102: 2832–2837. 10.1073/pnas.0409853102 15689398 PMC549489

[87] Sawyer SL, Emerman M, Malik HS. Ancient adaptive evolution of the primate antiviral DNA-editing enzyme APOBEC3G. PLoS Biol. 2004; 2: E275 10.1371/journal.pbio.0020275 15269786 PMC479043

[88] McNatt MW, Zang T, Hatziioannou T, Bartlett M, Fofana IB, Johnson WE, et al Species-Specific Activity of HIV-1 Vpu and Positive Selection of Tetherin Transmembrane Domain Variants. PLoS Pathog. 2009; 5: e1000300 10.1371/journal.ppat.1000300 19214216 PMC2633611

[89] Lim ES, Malik HS, Emerman M. Ancient adaptive evolution of tetherin shaped the functions of Vpu and Nef in human immunodeficiency virus and primate lentiviruses. J Virol. 2010; 84: 7124–7134. 10.1128/JVI.00468-10 20444900 PMC2898239

[90] Longdon B, Brockhurst MA, Russell CA, Welch JJ, Jiggins FM. The evolution and genetics of virus host shifts. PLoS Pathog. 2014; 10: e1004395 10.1371/journal.ppat.1004395 25375777 PMC4223060

[91] Woolhouse M, Scott F, Hudson Z, Howey R, Chase-Topping M. Human viruses: discovery and emergence. Phil Trans R Soc B. 2012; 367: 2864–2871. 10.1098/rstb.2011.0354 22966141 PMC3427559

[92] Li W, Wong S-K, Li F, Kuhn JH, Huang I-C, Choe H, et al Animal origins of the severe acute respiratory syndrome coronavirus: insight from ACE2-S-protein interactions. J Virol. 2006; 80: 4211–4219. 10.1128/JVI.80.9.4211-4219.2006 16611880 PMC1472041

[93] Fedeli C, Moreno H, Kunz S. Novel Insights into Cell Entry of Emerging Human Pathogenic Arenaviruses. J Mol Biol. 2018; 430: 1839–1852. 10.1016/j.jmb.2018.04.026 29705070

[94] Kailasan S, Agbandje-McKenna M, Parrish CR. Parvovirus Family Conundrum: What Makes a Killer? Annu Rev Virol. 2015; 2: 425–450. 10.1146/annurev-virology-100114-055150 26958923

[95] Arrildt KT, LaBranche CC, Joseph SB, Dukhovlinova EN, Graham WD, Ping L-H, et al Phenotypic Correlates of HIV-1 Macrophage Tropism. J Virol. 2015; 89: 11294–11311. 10.1128/JVI.00946-15 26339058 PMC4645658

[96] Dunfee RL, Thomas ER, Gorry PR, Wang J, Taylor J, Kunstman K, et al The HIV Env variant N283 enhances macrophage tropism and is associated with brain infection and dementia. Proc Natl Acad Sci USA. 2006;103: 15160–15165. 10.1073/pnas.0605513103 17015824 PMC1586182

[97] Laguette N, Sobhian B, Casartelli N, Ringeard M, Chable-Bessia C, Ségéral E, et al SAMHD1 is the dendritic- and myeloid-cell-specific HIV-1 restriction factor counteracted by Vpx. Nature. 2011; 474: 654–657. 10.1038/nature10117 21613998 PMC3595993

[98] Berger G, Durand S, Fargier G, Nguyen X-N, Cordeil S, Bouaziz S, et al APOBEC3A is a specific inhibitor of the early phases of HIV-1 infection in myeloid cells. PLoS Pathog. 2011; 7: e1002221 10.1371/journal.ppat.1002221 21966267 PMC3178557

[99] O'Brien WA, Namazi A, Kalhor H, Mao SH, Zack JA, Chen IS. Kinetics of human immunodeficiency virus type 1 reverse transcription in blood mononuclear phagocytes are slowed by limitations of nucleotide precursors. J Virol. 1994; 68: 1258–1263. 7507180 10.1128/jvi.68.2.1258-1263.1994 PMC236573

[100] Arfi V, Rivière L, Jarrosson-Wuillème L, Goujon C, Rigal D, Darlix J-L, et al Characterization of the early steps of infection of primary blood monocytes by human immunodeficiency virus type 1. J Virol. 2008; 82: 6557–6565. 10.1128/JVI.02321-07 18417568 PMC2447095

[101] Kilareski EM, Shah S, Nonnemacher MR, Wigdahl B. Regulation of HIV-1 transcription in cells of the monocyte-macrophage lineage. Retrovirology. 2009; 6: 118 10.1186/1742-4690-6-118 20030845 PMC2805609

[102] Joseph SB, Arrildt KT, Sturdevant CB, Swanstrom R. HIV-1 target cells in the CNS. J Neurovirol. 2015; 21: 276–289. 10.1007/s13365-014-0287-x 25236812 PMC4366351

[103] Schnell G, Joseph S, Spudich S, Price RW, Swanstrom R. HIV-1 replication in the central nervous system occurs in two distinct cell types. PLoS Pathog. 2011; 7: e1002286 10.1371/journal.ppat.1002286 22007152 PMC3188520

[104] Cherner M, Masliah E, Ellis RJ, Marcotte TD, Moore DJ, Grant I, et al Neurocognitive dysfunction predicts postmortem findings of HIV encephalitis. Neurology. 2002; 59: 1563–1567. 10.1212/01.wnl.0000034175.11956.79 12451198

[105] Antinori A, Arendt G, Becker JT, Brew BJ, Byrd DA, Cherner M, et al Updated research nosology for HIV-associated neurocognitive disorders. Neurology. 2007; 69: 1789–1799. 10.1212/01.WNL.0000287431.88658.8b 17914061 PMC4472366

[106] Cai Y, Sugimoto C, Arainga M, Midkiff CC, Liu DX, Alvarez X, et al Preferential Destruction of Interstitial Macrophages over Alveolar Macrophages as a Cause of Pulmonary Disease in Simian Immunodeficiency Virus-Infected Rhesus Macaques. J Immunol. 2015; 195: 4884–4891. 10.4049/jimmunol.1501194 26432896 PMC4637238

[107] Crowe SM, Westhorpe CLV, Mukhamedova N, Jaworowski A, Sviridov D, Bukrinsky M. The macrophage: the intersection between HIV infection and atherosclerosis. J Leukoc Biol. 2010; 87: 589–598. 10.1189/jlb.0809580 19952353 PMC3085483

[108] Stremlau M, Owens CM, Perron MJ, Kiessling M, Autissier P, Sodroski J. The cytoplasmic body component TRIM5alpha restricts HIV-1 infection in Old World monkeys. Nature. 2004; 427: 848–853. 10.1038/nature02343 14985764

[109] Meyerson NR, Warren CJ, Vieira DASA, Diaz-Griferro F, Sawyer SL. Species-specific vulnerability of RanBP2 shaped the evolution of SIV as it transmitted in African apes. PLoS Pathog. 2018;14: e1006906 10.1371/journal.ppat.1006906 29518153 PMC5843284

[110] Stabell AC, Hawkins J, Li M, Gao X, David M, Press WH, et al Non-human Primate Schlafen11 Inhibits Production of Both Host and Viral Proteins. PLoS Pathog. 2016; 12: e1006066 10.1371/journal.ppat.1006066 28027315 PMC5189954

[111] Nisole S, Lynch C, Stoye JP, Yap MW. A Trim5-cyclophilin A fusion protein found in owl monkey kidney cells can restrict HIV-1. Proc Natl Acad Sci USA. 2004; 101: 13324–13328. 10.1073/pnas.0404640101 15326303 PMC516566

[112] Sayah DM, Sokolskaja E, Berthoux L, Luban J. Cyclophilin A retrotransposition into TRIM5 explains owl monkey resistance to HIV-1. Nature. 2004; 430: 569–573. 10.1038/nature02777 15243629

[113] Cyranoski D. Monkey kingdom. Nature. 2016 532 (7599): 300–302. 10.1038/532300a 27111614

[114] Meyerson NR, Zhou L, Guo YR, Zhao C, Tao YJ, Krug RM, et al Nuclear TRIM25 Specifically Targets Influenza Virus Ribonucleoproteins to Block the Onset of RNA Chain Elongation. Cell Host Microbe. 2017; 22: 627–638.e7. 10.1016/j.chom.2017.10.003 29107643 PMC6309188

[115] Lou DI, Kim ET, Meyerson NR, Pancholi NJ, Mohni KN, Enard D, et al An Intrinsically Disordered Region of the DNA Repair Protein Nbs1 Is a Species-Specific Barrier to Herpes Simplex Virus 1 in Primates. Cell Host Microbe. 2016; 20: 178–188. 10.1016/j.chom.2016.07.003 27512903 PMC4982468

[116] Stabell AC, Meyerson NR, Gullberg RC, Gilchrist AR, Webb KJ, Old WM, et al Dengue viruses cleave STING in humans but not in nonhuman primates, their presumed natural reservoir. eLife. 2018; 7: e01081–16. 10.7554/eLife.31919 29557779 PMC5860865

[117] Zhang J, Zhao J, Xu S, Li J, He S, Zeng Y, et al Species-Specific Deamidation of cGAS by Herpes Simplex Virus UL37 Protein Facilitates Viral Replication. Cell Host Microbe. 2018; 24: 234–248.e5. 10.1016/j.chom.2018.07.004 30092200 PMC6094942

[118] Long EM, Rainwater SMJ, Lavreys L, Mandaliya K, Overbaugh J. HIV type 1 variants transmitted to women in Kenya require the CCR5 coreceptor for entry, regardless of the genetic complexity of the infecting virus. AIDS Res Hum Retroviruses. 2002; 18: 567–576. 10.1089/088922202753747914 12036486

[119] Aricescu AR, Lu W, Jones EY. A time- and cost-efficient system for high-level protein production in mammalian cells. Acta Crystallogr D Biol Crystallogr. 2006; 62: 1243–1250. 10.1107/S0907444906029799 17001101

[120] National Research Council (U.S.). Committee for the update of the Guide for the Care and Use of Laboratory Animals., Institute or Laboratory Animal Research (U.S.), National Academies Press (U.S.) 2011 Guide for the care and use of laboratory animals, 8th ed 10.1258/la.2010.010031

