# Supplementary figures and images for: Selective use of primate CD4 receptors by HIV-1
Source: PLoS Biol. 2019 Jun 10;17(6):e3000304. doi: 10.1371/journal.pbio.3000304 (PMC6586362; doi:10.1371/journal.pbio.3000304)

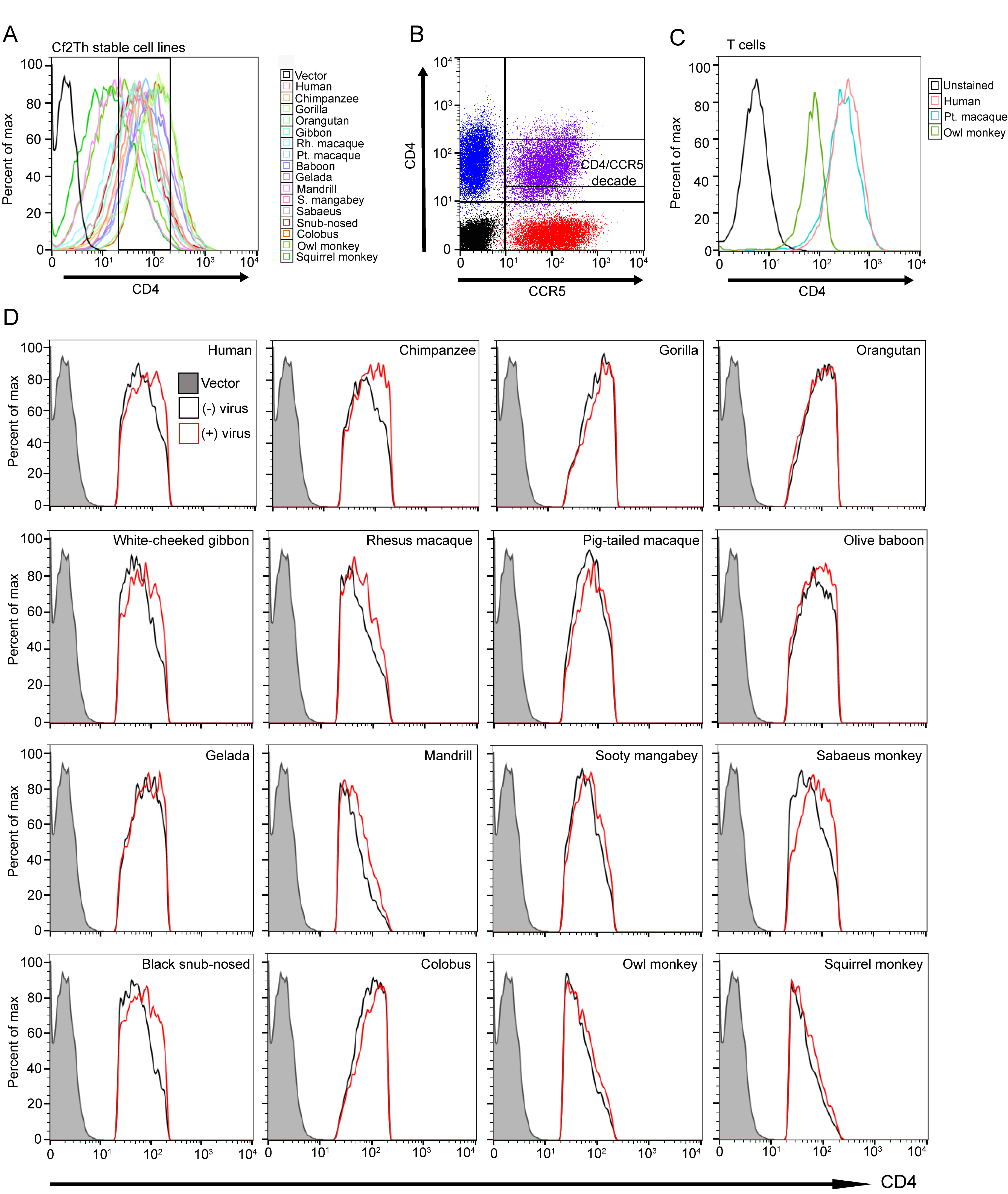

Supplement: S1 Fig — (A) Histograms of CD4 expression levels in Cf2Th cell lines engineered to stably express each primate CD4 receptor with human CCR5. (B) Gating strategy for enumerating the GFP+ (infected) cell population. Single stains of human CD4 (blue) and human CCR5 (red) as well as unstained cells (black) were used to denote receptor expression quadrants. A CD4 window was drawn such that it would capture equivalent CD4/CCR5 receptor expression across all stable cell lines shown in (A). GFP+ cells were enumerated within this window. (C) Surface expression of CD4 from human T cells (Hut-78 cells) and primate T cells that we isolated directly from primate blood. (D) Histograms of CD4 expression levels in Cf2Th cell lines with (red line) and without (black line) HIV-1 infection. Shaded histograms are from cells transduced with an empty vector control and denote the CD4-negative population. Data associated with this figure can be found in the supplemental data file (S7 Data). CCR5, C-C motif chemokine receptor 5; GFP, green fluorescent protein. (TIF) [file pbio.3000304.s001.tif]

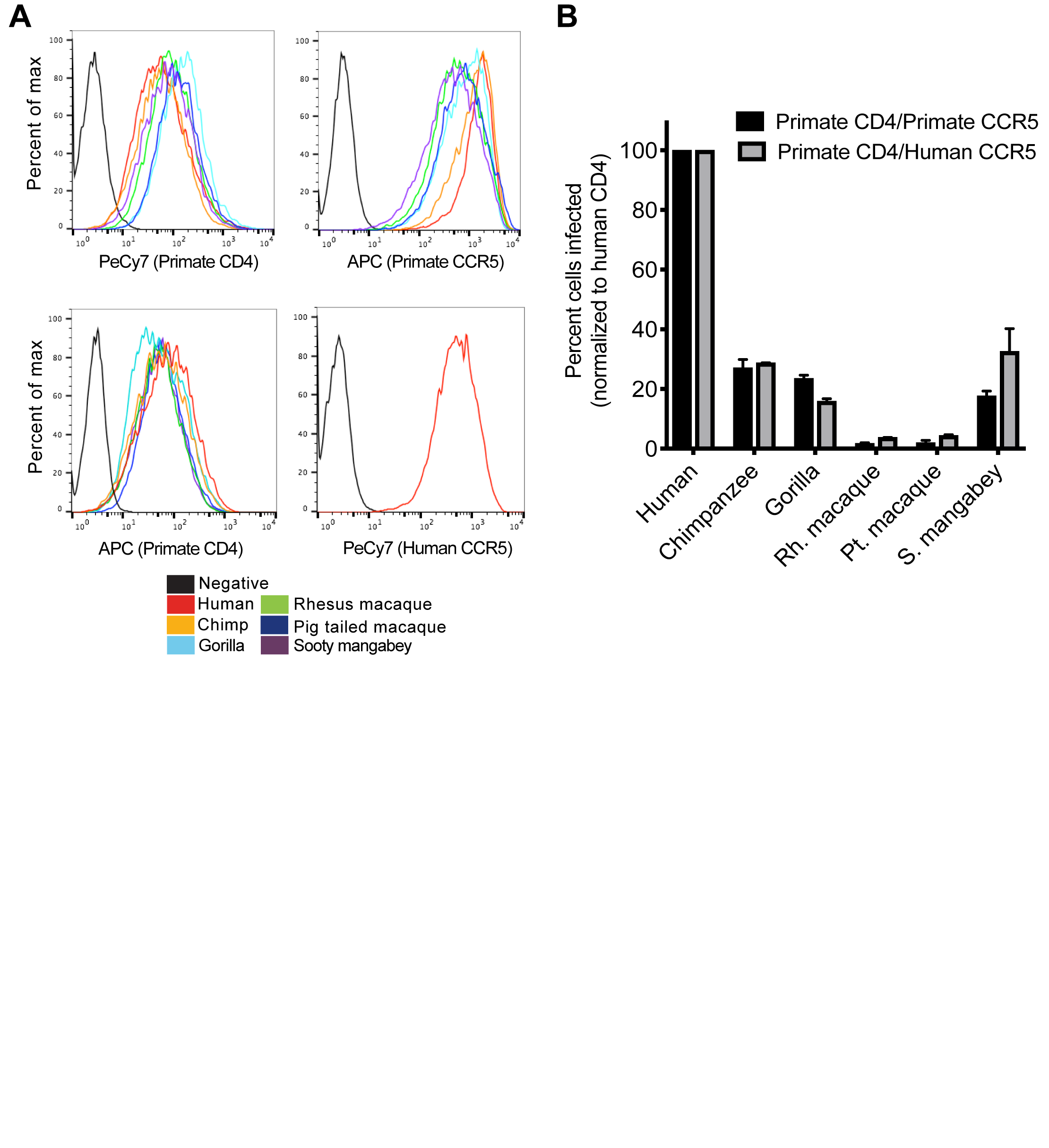

Supplement: S2 Fig — (A) Histograms of CD4 and CCR5 expression levels in Cf2Th cell lines made to stably express primate CD4/CCR5 receptor pairs from each primate species (top) or each primate CD4 paired with human CCR5 (bottom). (B) Cf2Th cell lines stably expressing primate CD4/CCR5 receptor pairs from each primate species (black bars) and each primate CD4 paired with human CCR5 (gray bars) were infected with HIV-1 GFP pseudotyped with a subtype A Envelope (BG505). Error bars represent the mean + SEM from two independent experiments, each with three technical replicates. Data associated with this figure can be found in the supplemental data file (S8 and S9 Datas). CCR5, C-C motif chemokine receptor 5; GFP, green fluorescent protein. (TIF) [file pbio.3000304.s002.tif]

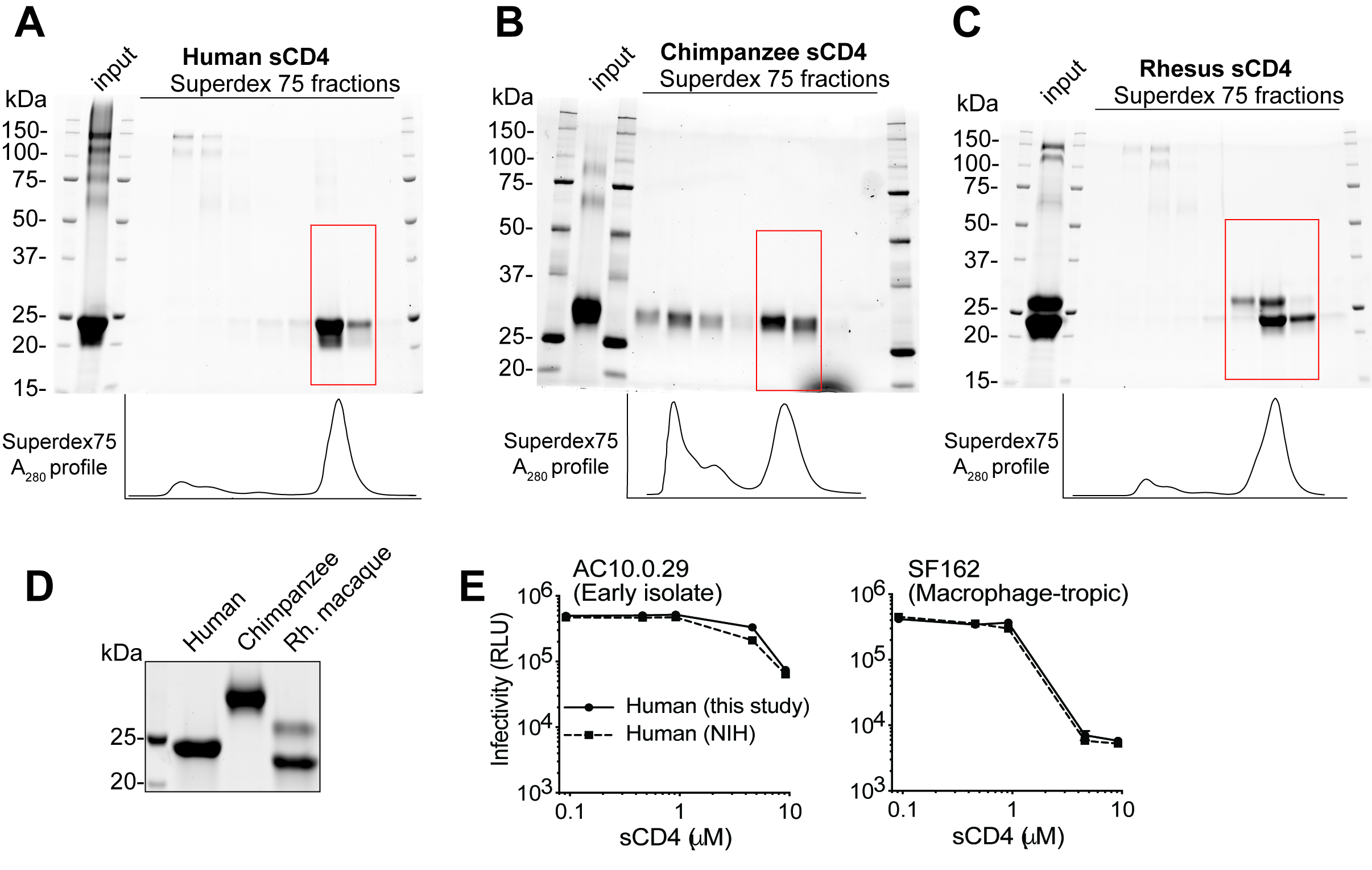

Supplement: S3 Fig — (A, B, C) Size-exclusion profiles of (A) human, (B) chimpanzee, and (C) rhesus macaque soluble CD4 proteins (sCD4). The input sample is made from combined fractions eluted from the Ni-NTA column. Total protein was visualized using the TGX stain-free system (Bio-Rad). Fractions collected from the Superdex 75 column are each 1 ml. Fractions indicated with a red box eluted at a volume consistent with the molecular weight of sCD4 monomers and were combined for use in downstream experiments. Plots below the gels are A280 absorbance readings from the FPLC spectrophotometer. (D) Total protein stain of purified sCD4 molecules. Human, chimpanzee, and rhesus macaque CD4 are all differentially glycosylated, explaining the differences in migration [48]. (E) HIV-1 pseudotyped with the indicated Envs (top of graphs), was preincubated with increasing concentrations of human sCD4 produced in this study (solid line; see panel A) or a commercially available sCD4 obtained from National Institutes of Health AIDS Reagent Program (#7356) (dashed line), and then used to infect TZM-bl cells. Error bars represent the SD from n = 4 technical replicates. Data associated with this figure can be found in the supplemental data file (S10 Data). FPLC, fast protein liquid chromatography; TGX, Tris-Glycine eXtended (TIF) [file pbio.3000304.s003.tif]

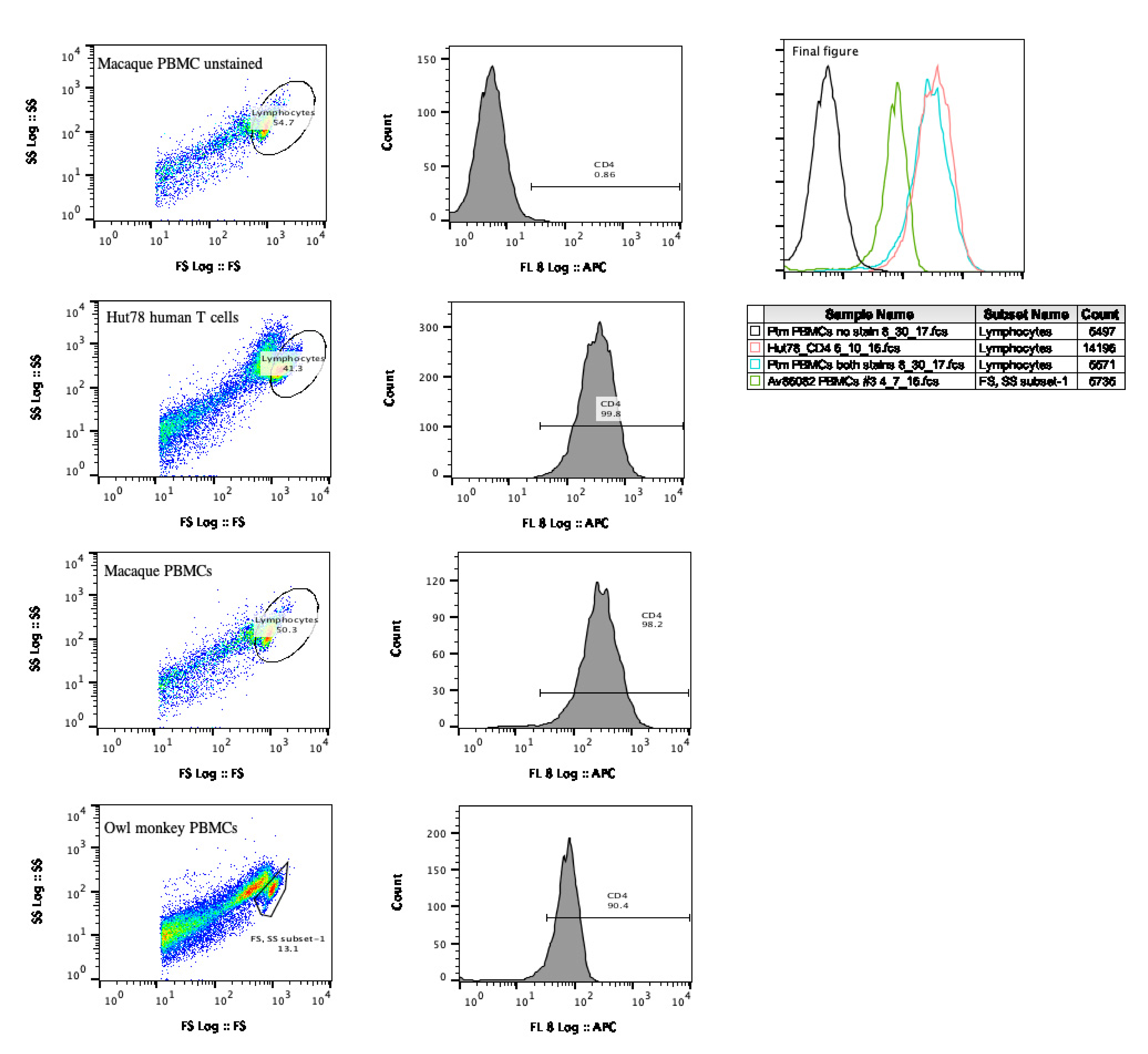

Supplement: S7 Data — (ZIP) [file pbio.3000304.s011.zip › S1 Fig/S1 C/S1 C.tif]
