# Supplementary material for: Selective use of primate CD4 receptors by HIV-1
Source: PLoS Biol. 2019 Jun 10;17(6):e3000304. doi: 10.1371/journal.pbio.3000304 (PMC6586362; doi:10.1371/journal.pbio.3000304)
Supplement: S1 Table — (DOCX) [file pbio.3000304.s004.docx]

**S1 Table. Envelope clones used in this study.**

|  | Env clone | Subtype | Country of origin | Estimated time post infection | Risk factor | Specimen source^a^ | Infection status^b^ | References | NIH ARP catalog # |
| --- | --- | --- | --- | --- | --- | --- | --- | --- | --- |
| Macrophage-tropic | SF162 | B | USA | AIDS | NA | Brain | Chronic | [64-67] | 10463 |
|  | YU2 | B | USA | AIDS | NA | Brain | Chronic | [31,68] | - |
|  | BaL | B | USA | AIDS | NA | Lungs | Chronic | [69,70] | 11446 |
|  | 89.6 | B | USA | AIDS | NA | ccPBMC | Chronic | [71,72] | 12485 |
|  | | | | | | | | | |
| Chronic isolates | CNE55 | CRF01_AE | China | >6 months | IVDU | PBMC | Chronic | [49,121] | 12661 |
|  | CH119 | CRF07_BC | China | >6 months | IVDU | ccPBMC | Chronic | [49] | 12659 |
|  | CNE8 | CRF01_AE | China | >6 months | IVDU | PBMC | Chronic | [49,121] | 12653 |
|  | X1632 | G | Spain | >6 months | Heterosexual | Plasma | Chronic | [49,122] | 12656 |
|  | | | | | | | | | |
| Patient matched early and chronic pairs | CH40 | B | USA | 23 days | MSM | serum/plasma | Early | [3,14] | - |
|  | CH40.6mo |  |  | 6 months |  | consensus | 6-month | [14] | - |
|  | CH470 | B | USA | N/A | MSM | PBMC | Early | [14,50] | - |
|  | CH470.6mo |  |  | 6 months |  | consensus | 6-month | [14] | - |
|  | CH58 | B | USA | 36 days | MSM | serum/plasma | Early | [3,14] | - |
|  | CH58.6mo |  |  | 6 months |  | consensus | 6-month | [14,51] | - |
|  | CH77 | B | USA | 35 days | MSM | serum/plasma | Early | [3,14] | - |
|  | CH77.6mo |  |  | 6 months |  | consensus | 6-month | [14,51] | - |
| Maternal-infant pairs | BF535.W6M.A1 | D/A | Kenya | <6 weeks | Delivery or breastfeeding | PBMC | Early | [9] | 11517 |
|  | MF535.W0M.C1 |  |  | N/A | N/A | PBMC | Chronic | [9] | 11526 |
|  | BG505.W6M.C2 | A | Kenya | <6 weeks | Delivery or breastfeeding | PBMC | Early | [9] | 11518 |
|  | MG505.W0M.A2 |  |  | N/A | N/A | PBMC | Chronic | [9] | 11528 |
|  | BJ613.W6M.E1 | A | Kenya | <6 weeks | Delivery or breastfeeding  Maternal-infant pairs | PBMC | Early | [9] | 11521 |
|  | MJ613.W0M.A2 |  |  | N/A | N/A | PBMC | Chronic | [9] | 11534 |
|  | BL274.W6M.A3 | A | Kenya | <6 weeks | Delivery or breastfeeding | PBMC | Early | [9] | 11525 |
|  | ML274.W0M.B1 |  |  | N/A | N/A | PBMC | Chronic | [9] | 11540 |
|  | BK184.W6M.D2 | C/D | Kenya | <6 weeks | Delivery or breastfeeding | PBMC | Early | [9] | 11522 |
|  | MK184.W0M.E4 |  |  | N/A | N/A | PBMC | Chronic | [9] | 11536 |
|  | BJ412.W6M.S3 | C | Kenya | <6 weeks | Delivery or breastfeeding | PBMC | Early | [9] | 11520 |
|  | MJ412.W0M.B1 |  |  | N/A | N/A | PBMC | Chronic | [9] | 11532 |
|  | BI206.W6P.A1 | A | Kenya | <6 weeks | Delivery or breastfeeding | PBMC | TF | [9] | 11519 |
|  | MI206.W0M.B1 |  |  | N/A | N/A | PBMC | Chronic | [9] | 11530 |
|  | | | | | | | | | |
| Global HIV-1 isolates | BG505.W6M.B1 | A | Kenya | <6 weeks | Delivery or breastfeeding | PBMC | Early | [9] | - |
|  | QF495.23M.A1 | A | Kenya | 23 days | Heterosexual | PBMC | Early | [52] | 11890 |
|  | AC10.0.29 | B | USA | 4 weeks | MSM | ccPMBC | Early | [53] | 11024 |
|  | CAP210.2.00.E8 | C | South Africa | 5 weeks | Heterosexual | Plasma | Early | [36] | 11317 |
|  | QB857.23I.B3 | D | Kenya | 110 days | Heterosexual | PBMC | Early | [52] | 11915 |
| Us  Used in cell-cell fusion assays | ZM53M.PB12 | C | Zambia | <12 weeks | Heterosexual | PBMC | Early | [5] | 11313 |
|  | 6535.3 | B | USA | 8 weeks | MSM | ccPBMC | Early | [53] | 11017 |
|  | SC422661.8 | B | Trinidad | 4 weeks | Heterosexual | Plasma | Early | [53] | 11058 |
|  | PVO.4 | B | Italy | 4 weeks | MSM | ccPBMC | Early | [53] | 11022 |
|  | RHPA4259.7 | B | USA | <8 weeks | Heterosexual | Plasma | Early | [53] | 11036 |
|  | REJO4541.67 | B | USA | 2 weeks | Heterosexual | Plasma | Early | [53] | 11035 |
|  | WITO4160.33 | B | USA | 1 week | Heterosexual | Plasma | Early | [53] | 11033 |
|  | QD435.100M.A4 | D | Kenya | 100 days | Heterosexual | PBMC | Early | [52,118] | 11917 |
|  | Q23.ENV.17 | A | Kenya | ~1 year | Heterosexual | PBMC | Chronic | [42,123] | 11547 |
|  | QG984.21M.A3 | A | Kenya | 21 days | Heterosexual | PBMC | Early | [52,118] | 11894 |
|  | QH209.14M.A2 | A | Kenya | 14 days | Heterosexual | PBMC | Early | [52,118] | 11895 |
|  | QH359.21M.E2 | A | Kenya | 21 days | Heterosexual | PBMC | Early | [52,118] | 11900 |
|  | ZM109F.PB4 | C | Zambia | <12 weeks | Heterosexual | PBMC | Early | [5] | 11314 |
|  | QB726.70M.B3 | A | Kenya | 70 days | Heterosexual | PBMC | Early | [52,118] | 11889 |
|  | QA790.204I.A4 | A/D | Kenya | 204 days | Heterosexual | ccPBMC | Chronic | [52,118] | 11901 |
|  | Du156.12 | C | South Africa | <4 weeks | Heterosexual | ccPBMC | Early | [52,118] | 11306 |
|  | Du422.1 | C | South Africa | 8 weeks | Heterosexual | ccPBMC | Early | [118] | 11308 |
|  | CAP45.2.00.G3 | C | South Africa | 5 weeks | Heterosexual | Plasma | Early | [118] | 11316 |
|  | ZM135M.PL10a | C | Zambia | <15 weeks | Heterosexual | Plasma | Early | [5] | 11315 |
|  | QG393.60M.A1 | A2/D | Kenya | 60 days | Heterosexual | PBMC | Early | [52,118] | 11905 |
|  | QH343.21M.A10 | A | Kenya | 21 days | Heterosexual | PBMC | Early | [52,118] | 11896 |
|  | QH343.21M.B5  Used in cell-cell fusion assays | Subclone of patient QH343 described above | | | |  |  |  |  |
|  | QA013.70I.H1 | D | Kenya | 70 days | Heterosexual | ccPBMC | Early | [52,118] | 11911 |
|  | QA013.70I.M12 | Subclone of patient QA013 described above | | | |  |  |  |  |
|  | QA465.59M.A1 | D | Kenya | 59 days | Heterosexual | PBMC | Early | [52,118] | 11913 |
|  | QA465.59M.D1 | Subclone of patient QA465 described above | | | |  |  |  |  |
|  | QF495.23M.A1 | Described above | | | | | | | |
|  | QF495.23M.B2 | Subclone of patient QF495 described above | | | |  |  |  |  |
|  | QF495.23M.A3 | Subclone of patient QF495 described above | | | |  |  |  |  |
|  | QF495.23M.D1 | Subclone of patient QF495 described above | | | |  |  |  |  |
|  | AC10.0.29 | Described above | |  |  |  |  |  |  |
|  | QB857.23I.B3 | Described above | |  |  |  |  |  |  |
|  | BG505.W6M.B1 | Described above | |  |  |  |  |  |  |

^a^ ccPBMC: derived from co-culture of infected and uninfected PBMCs

^b^ Early isolates are classified as <150 days post infection while chronic isolates are >150 days as described in the text.

**References**

1. Sagar M. HIV-1 transmission biology: selection and characteristics of infecting viruses. J Infect Dis. 2010; 202 Suppl 2: S289–96.

2. Kearney M, Maldarelli F, Shao W, Margolick JB, Daar ES, Mellors JW, et al. Human Immunodeficiency Virus Type 1 Population Genetics and Adaptation in Newly Infected Individuals. J Virol. 2009; 83: 2715–2727. doi:10.1128/JVI.01960-08

3. Keele BF, Giorgi EE, Salazar-Gonzalez JF, Decker JM, Pham KT, Salazar MG, et al. Identification and characterization of transmitted and early founder virus envelopes in primary HIV-1 infection. Proc Natl Acad Sci USA. 2008; 105: 7552–7557.

4. Abrahams MR, Anderson JA, Giorgi EE, Seoighe C, Mlisana K, Ping LH, et al. Quantitating the multiplicity of infection with human immunodeficiency virus type 1 subtype C reveals a non-poisson distribution of transmitted variants. J Virol. 2009; 83: 3556–3567.

5. Derdeyn CA, Decker JM, Bibollet-Ruche F, Mokili JL, Muldoon M, Denham SA, et al. Envelope-constrained neutralization-sensitive HIV-1 after heterosexual transmission. Science. 2004; 303: 2019–2022. doi:10.1126/science.1093137

6. Tully DC, Ogilvie CB, Batorsky RE, Bean DJ, Power KA, Ghebremichael M, et al. Differences in the Selection Bottleneck between Modes of Sexual Transmission Influence the Genetic Composition of the HIV-1 Founder Virus. PLoS Pathog. 2016; 12: e1005619. doi:10.1371/journal.ppat.1005619

7. Bar KJ, Li H, Chamberland A, Tremblay C, Routy JP, Grayson T, et al. Wide variation in the multiplicity of HIV-1 infection among injection drug users. J Virol. 2010; 84: 6241–6247. doi:10.1128/JVI.00077-10

8. Masharsky AE, Dukhovlinova EN, Verevochkin SV, Toussova OV, Skochilov RV, Anderson JA, et al. A Substantial Transmission Bottleneck among Newly and Recently HIV-1-Infected Injection Drug Users in St Petersburg, Russia. J Infect Dis. 2010; 201: 1697–1702. doi:10.1086/652702

9. Wu X, Parast AB, Richardson BA, Nduati R, John-Stewart G, Mbori-Ngacha D, et al. Neutralization escape variants of human immunodeficiency virus type 1 are transmitted from mother to infant. J Virol. 2006; 80: 835–844. doi:10.1128/JVI.80.2.835-844.2006

10. Parker ZF, Iyer SS, Wilen CB, Parrish NF, Chikere KC, Lee FH, et al. Transmitted/Founder and Chronic HIV-1 Envelope Proteins Are Distinguished by Differential Utilization of CCR5. J Virol. 2013; 87: 2401–2411. doi:10.1128/JVI.02964-12

11. Go EP, Hewawasam G, Liao H-X, Chen H, Ping L-H, Anderson JA, et al. Characterization of glycosylation profiles of HIV-1 transmitted/founder envelopes by mass spectrometry. J Virol. 2011; 85: 8270–8284. doi:10.1128/JVI.05053-11

12. Liu Y, Curlin ME, Diem K, Zhao H, Ghosh AK, Zhu H, et al. Env length and N-linked glycosylation following transmission of human immunodeficiency virus Type 1 subtype B viruses. Virology. 2008; 374: 229–233. doi:10.1016/j.virol.2008.01.029

13. Liao HX, Tsao CY, Alam SM, Muldoon M, Vandergrift N, Ma BJ, et al. Antigenicity and Immunogenicity of Transmitted/Founder, Consensus, and Chronic Envelope Glycoproteins of Human Immunodeficiency Virus Type 1. J Virol. 2013; 87: 4185–4201. doi:10.1128/JVI.02297-12

14. Foster TL, Wilson H, Iyer SS, Coss K, Doores K, Smith S, et al. Resistance of Transmitted Founder HIV-1 to IFITM-Mediated Restriction. Cell Host Microbe. 2016; 20: 429–442. doi:10.1016/j.chom.2016.08.006

15. Fenton-May AE, Dibben O, Emmerich T, Ding H, Pfafferott K, Aasa-Chapman MM, et al. Relative resistance of HIV-1 founder viruses to control by interferon-alpha. Retrovirology. 2013; 10: 146. doi:10.1186/1742-4690-10-146

16. Warren CJ, Sawyer SL. How host genetics dictates successful viral zoonosis. PLoS Biol. 2019; 17: e3000217. doi:10.1371/journal.pbio.3000217

17. Sharp PM, Hahn BH. Origins of HIV and the AIDS pandemic. Cold Spring Harb Perspect Med. 2011; 1: a006841. doi:10.1101/cshperspect.a006841

18. Hatziioannou T, Evans DT. Animal models for HIV/AIDS research. Nat Rev Micro. 2012; 10: 852–867. doi:10.1038/nrmicro2911

19. Bell SM, Bedford T. Modern-day SIV viral diversity generated by extensive recombination and cross-species transmission. PLoS Pathog. 2017;13: e1006466. doi:10.1371/journal.ppat.1006466

20. Zhang ZD, Weinstock G, Gerstein M. Rapid evolution by positive Darwinian selection in T-cell antigen CD4 in primates. J Mol Evol. 2008; 66: 446–456. doi:10.1007/s00239-008-9097-1

21. Meyerson NR, Rowley PA, Swan CH, Le DT, Wilkerson GK, Sawyer SL. Positive selection of primate genes that promote HIV-1 replication. Virology. 2014; 454-455: 291–298. doi:10.1016/j.virol.2014.02.029

22. Demogines A, Abraham J, Choe H, Farzan M, Sawyer SL. Dual host-virus arms races shape an essential housekeeping protein. PLoS Biol. 2013; 11: e1001571. doi:10.1371/journal.pbio.1001571

23. Demogines A, Farzan M, Sawyer SL. Evidence for ACE2-utilizing coronaviruses (CoVs) related to severe acute respiratory syndrome CoV in bats. J Virol. 2012; 86: 6350–6353. doi:10.1128/JVI.00311-12

24. Kaelber JT, Demogines A, Harbison CE, Allison AB, Goodman LB, Ortega AN, et al. Evolutionary reconstructions of the transferrin receptor of Caniforms supports canine parvovirus being a re-emerged and not a novel pathogen in dogs. PLoS Pathog. 2012; 8: e1002666. doi:10.1371/journal.ppat.1002666

25. Kerr SA, Jackson EL, Lungu OI, Meyer AG, Demogines A, Ellington AD, et al. Computational and Functional Analysis of the Virus-Receptor Interface Reveals Host Range Trade-Offs in New World Arenaviruses. J Virol. 2015; 89: 11643–11653. doi:10.1128/JVI.01408-15

26. Martin C, Buckler-White A, Wollenberg K, Kozak CA. The avian XPR1 gammaretrovirus receptor is under positive selection and is disabled in bird species in contact with virus-infected wild mice. J Virol. 2013; 87: 10094–10104. doi:10.1128/JVI.01327-13

27. Meyerson NR, Sawyer SL. Two-stepping through time: mammals and viruses. Trends Microbiol. 2011; 19: 286–294.

28. Liu Q, Acharya P, Dolan MA, Zhang P, Guzzo C, Lu J, et al. Quaternary contact in the initial interaction of CD4 with the HIV-1 envelope trimer. Nat Struct Mol Biol. 2017; 24: 370–378. doi:10.1038/nsmb.3382

29. Joseph SB, Swanstrom R. The evolution of HIV-1 entry phenotypes as a guide to changing target cells. J Leukoc Biol. 2018; 103: 421–431. doi:10.1002/JLB.2RI0517-200R

30. Humes D, Overbaugh J. Adaptation of Subtype A Human Immunodeficiency Virus Type 1 Envelope to Pig-Tailed Macaque Cells. J Virol. 2011; 85: 4409–4420. doi:10.1128/JVI.02244-10

31. Humes D, Emery S, Laws E, Overbaugh J. A Species-Specific Amino Acid Difference in the Macaque CD4 Receptor Restricts Replication by Global Circulating HIV-1 Variants Representing Viruses from Recent Infection. J Virol. 2012; 86: 12472–12483.

32. Del Prete GQ, Ailers B, Moldt B, Keele BF, Estes JD, Rodriguez A, et al. Selection of Unadapted, Pathogenic SHIVs Encoding Newly Transmitted HIV-1 Envelope Proteins. Cell Host Microbe. 2014; 16: 412–418. doi:10.1016/j.chom.2014.08.003

33. Meyerson NR, Sharma A, Wilkerson GK, Overbaugh J, Sawyer SL. Identification of Owl Monkey CD4 Receptors Broadly Compatible with Early-Stage HIV-1 Isolates. J Virol. 2015; 89: 8611–8622. doi:10.1128/JVI.00890-15

34. Cohen MS, Gay CL, Busch MP, Hecht FM. The detection of acute HIV infection. J Infect Dis. 2010; 202 Suppl 2: S270–7. doi:10.1086/655651

35. Fiebig EW, Wright DJ, Rawal BD, Garrett PE, Schumacher RT, Peddada L, et al. Dynamics of HIV viremia and antibody seroconversion in plasma donors: implications for diagnosis and staging of primary HIV infection. AIDS. 2003; 17: 1871–1879. doi:10.1097/01.aids.0000076308.76477.b8

36. Li M, Salazar-Gonzalez JF, Derdeyn CA, Morris L, Williamson C, Robinson JE, et al. Genetic and neutralization properties of subtype C human immunodeficiency virus type 1 molecular env clones from acute and early heterosexually acquired infections in Southern Africa. J Virol. 2006;80: 11776–11790. doi:10.1128/JVI.01730-06

37. Etemad B, Fellows A, Kwambana B, Kamat A, Feng Y, Lee S, et al. Human immunodeficiency virus type 1 V1-to-V5 envelope variants from the chronic phase of infection use CCR5 and fuse more efficiently than those from early after infection. J Virol. 2009; 83: 9694–9708. doi:10.1128/JVI.00925-09

38. Wang H, Cohen AA, Galimidi RP, Gristick HB, Jensen GJ, Bjorkman PJ. Cryo-EM structure of a CD4-bound open HIV-1 envelope trimer reveals structural rearrangements of the gp120 V1V2 loop. Proc Natl Acad Sci USA. 2016; 113: E7151–E7158. doi:10.1073/pnas.1615939113

39. Sanders RW, Derking R, Cupo A, Julien J-P, Yasmeen A, de Val N, et al. A next-generation cleaved, soluble HIV-1 Env trimer, BG505 SOSIP.664 gp140, expresses multiple epitopes for broadly neutralizing but not non-neutralizing antibodies. PLoS Pathog. 2013; 9: e1003618. doi:10.1371/journal.ppat.1003618

40. Sanders RW, Moore JP. Native-like Env trimers as a platform for HIV-1 vaccine design. Immunol Rev. 2017; 275: 161–182. doi:10.1111/imr.12481

41. Lu M, Ma X, Castillo-Menendez LR, Gorman J, Alsahafi N, Ermel U, et al. Associating HIV-1 envelope glycoprotein structures with states on the virus observed by smFRET. Nature. 2019; 568: 415–419. doi:10.1038/s41586-019-1101-y

42. Poss M, Overbaugh J. Variants from the diverse virus population identified at seroconversion of a clade A human immunodeficiency virus type 1-infected woman have distinct biological properties. J Virol. 1999; 73: 5255–5264.

43. Ryu SE, Kwong PD, Truneh A, Porter TG, Arthos J, Rosenberg M, et al. Crystal structure of an HIV-binding recombinant fragment of human CD4. Nature. 1990; 348: 419–426. doi:10.1038/348419a0

44. Wang JH, Yan YW, Garrett TP, Liu JH, Rodgers DW, Garlick RL, et al. Atomic structure of a fragment of human CD4 containing two immunoglobulin-like domains. Nature. 1990; 348: 411–418. doi:10.1038/348411a0

45. Kwong PD, Wyatt R, Robinson J, Sweet RW, Sodroski J, Hendrickson WA. Structure of an HIV gp120 envelope glycoprotein in complex with the CD4 receptor and a neutralizing human antibody. Nature. 1998; 393: 648–659. doi:10.1038/31405

46. Arthos J, Deen KC, Chaikin MA, Fornwald JA, Sathe G, Sattentau QJ, et al. Identification of the residues in human CD4 critical for the binding of HIV. Cell. 1989; 57: 469–481.

47. Landau NR, Warton M, Littman DR. The envelope glycoprotein of the human immunodeficiency virus binds to the immunoglobulin-like domain of CD4. Nature. 1988; 334: 159–162. doi:10.1038/334159a0

48. Warren CJ, Meyerson NR, Stabell AC, Fattor WT, Wilkerson GK, Sawyer SL. A glycan shield on chimpanzee CD4 protects against infection by primate lentiviruses (HIV/SIV). Proc Natl Acad Sci USA. 2019; 116: 3229-3238. doi: 10.1073/pnas.1821197116

49. deCamp A, Hraber P, Bailer RT, Seaman MS, Ochsenbauer C, Kappes J, et al. Global panel of HIV-1 Env reference strains for standardized assessments of vaccine-elicited neutralizing antibodies. J Virol. 2014;88: 2489–2507. doi:10.1128/JVI.02853-13

50. Parrish NF, Gao F, Li H, Giorgi EE, Barbian HJ, Parrish EH, et al. Phenotypic properties of transmitted founder HIV-1. Proc Natl Acad Sci USA. 2013;110: 6626–6633.

51. Freel SA, Picking RA, Ferrari G, Ding H, Ochsenbauer C, Kappes JC, et al. Initial HIV-1 antigen-specific CD8+ T cells in acute HIV-1 infection inhibit transmitted/founder virus replication. J Virol. 2012; 86: 6835–6846. doi:10.1128/JVI.00437-12

52. Blish CA, Jalalian-Lechak Z, Rainwater S, Nguyen M-A, Dogan OC, Overbaugh J. Cross-subtype neutralization sensitivity despite monoclonal antibody resistance among early subtype A, C, and D envelope variants of human immunodeficiency virus type 1. J Virol. 2009; 83: 7783–7788. doi:10.1128/JVI.00673-09

53. Li M, Gao F, Mascola JR, Stamatatos L, Polonis VR, Koutsoukos M, et al. Human immunodeficiency virus type 1 env clones from acute and early subtype B infections for standardized assessments of vaccine-elicited neutralizing antibodies. J Virol. 2005; 79: 10108–10125. doi:10.1128/JVI.79.16.10108-10125.2005

54. Swanstrom R, Graham WD, Zhou S. Sequencing the Biology of Entry: The Retroviral env Gene. Curr Top Microbiol Immunol. 2017; 407: 65–82. doi:10.1007/82_2017_35

55. Ping L-H, Joseph SB, Anderson JA, Abrahams M-R, Salazar-Gonzalez JF, Kincer LP, et al. Comparison of viral Env proteins from acute and chronic infections with subtype C human immunodeficiency virus type 1 identifies differences in glycosylation and CCR5 utilization and suggests a new strategy for immunogen design. J Virol. 2013; 87: 7218–7233. doi:10.1128/JVI.03577-12

56. Parrish NF, Wilen CB, Banks LB, Iyer SS, Pfaff JM, Salazar-Gonzalez JF, et al. Transmitted/founder and chronic subtype C HIV-1 use CD4 and CCR5 receptors with equal efficiency and are not inhibited by blocking the integrin α4β7. PLoS Pathog. 2012; 8: e1002686. doi:10.1371/journal.ppat.1002686

57. Sheppard HW, Celum C, Michael NL, O'Brien S, Dean M, Carrington M, et al. HIV-1 infection in individuals with the CCR5-Delta32/Delta32 genotype: acquisition of syncytium-inducing virus at seroconversion. J AIDS. 2002; 29: 307–313.

58. Oh D-Y, Jessen H, Kücherer C, Neumann K, Oh N, Poggensee G, et al. CCR5Delta32 genotypes in a German HIV-1 seroconverter cohort and report of HIV-1 infection in a CCR5Delta32 homozygous individual. PLoS ONE. 2008; 3: e2747. doi:10.1371/journal.pone.0002747

59. Huang W, Eshleman SH, Toma J, Stawiski E, Whitcomb JM, Jackson JB, et al. Vertical transmission of X4-tropic and dual-tropic HIV-1 in five Ugandan mother-infant pairs. AIDS. 2009; 23: 1903–1908. doi:10.1097/QAD.0b013e32832f1802

60. Huang W, Toma J, Stawiski E, Fransen S, Wrin T, Parkin N, et al. Characterization of human immunodeficiency virus type 1 populations containing CXCR4-using variants from recently infected individuals. AIDS Res Hum Retroviruses. 2009; 25: 795–802. doi:10.1089/aid.2008.0252

61. de Mendoza C, Rodriguez C, García F, Eiros JM, Ruiz L, Caballero E, et al. Prevalence of X4 tropic viruses in patients recently infected with HIV-1 and lack of association with transmission of drug resistance. J Antimicrob Chemother. 2007; 59: 698–704. doi:10.1093/jac/dkm012

62. Satomi M, Shimizu M, Shinya E, Watari E, Owaki A, Hidaka C, et al. Transmission of macrophage-tropic HIV-1 by breast-milk macrophages via DC-SIGN. J Infect Dis. 2005; 191: 174–181. doi:10.1086/426829

63. Peters PJ, Sullivan WM, Dueñas-Decamp MJ, Bhattacharya J, Ankghuambom C, Brown R, et al. Non-macrophage-tropic human immunodeficiency virus type 1 R5 envelopes predominate in blood, lymph nodes, and semen: implications for transmission and pathogenesis. J Virol. 2006; 80: 6324–32. doi:10.1128/JVI.02328-05

64. Cheng-Mayer C, Liu R, Landau NR, Stamatatos L. Macrophage tropism of human immunodeficiency virus type 1 and utilization of the CC-CKR5 coreceptor. J Virol. 1997; 71: 1657–1661.

65. Stamatatos L, Lim M, Cheng-Mayer C. Generation and structural analysis of soluble oligomeric gp140 envelope proteins derived from neutralization-resistant and neutralization-susceptible primary HIV type 1 isolates. AIDS Res Hum Retroviruses. 2000; 16: 981–994. doi:10.1089/08892220050058407

66. Stamatatos L, Wiskerchen M, Cheng-Mayer C. Effect of major deletions in the V1 and V2 loops of a macrophage-tropic HIV type 1 isolate on viral envelope structure, cell entry, and replication. AIDS Res Hum Retroviruses. 1998; 14: 1129–1139. doi:10.1089/aid.1998.14.1129

67. Cheng-Mayer C, Levy JA. Distinct biological and serological properties of human immunodeficiency viruses from the brain. Ann Neurol. 1988; 23: S58–S61.

68. Li Y, Hui H, Burgess CJ, Price RW, Sharp PM, Hahn BH, et al. Complete nucleotide sequence, genome organization, and biological properties of human immunodeficiency virus type 1 in vivo: evidence for limited defectiveness and complementation. J Virol. 1992; 66: 6587–6600.

69. Li Y, Svehla K, Mathy NL, Voss G, Mascola JR, Wyatt R. Characterization of antibody responses elicited by human immunodeficiency virus type 1 primary isolate trimeric and monomeric envelope glycoproteins in selected adjuvants. J Virol. 2006; 80: 1414–1426. doi:10.1128/JVI.80.3.1414-1426.2006

70. Gartner S, Markovits P, Markovitz DM, Kaplan MH, Gallo RC, Popovic M. The role of mononuclear phagocytes in HTLV-III/LAV infection. Science. 1986; 233: 215–219.

71. Carter CC, Onafuwa-Nuga A, McNamara LA, Riddell J, Bixby D, Savona MR, et al. HIV-1 infects multipotent progenitor cells causing cell death and establishing latent cellular reservoirs. Nat Med. 2010; 16: 446–451. doi:10.1038/nm.2109

72. Collman R, Balliet JW, Gregory SA, Friedman H, Kolson DL, Nathanson N, et al. An infectious molecular clone of an unusual macrophage-tropic and highly cytopathic strain of human immunodeficiency virus type 1. J Virol. 1992; 66: 7517–7521.

73. Salimi H, Roche M, Webb N, Gray LR, Chikere K, Sterjovski J, et al. Macrophage-tropic HIV-1 variants from brain demonstrate alterations in the way gp120 engages both CD4 and CCR5. J Leukoc Biol. 2013; 93: 113–126. doi:10.1189/jlb.0612308

74. Joseph SB, Arrildt KT, Swanstrom AE, Schnell G, Lee B, Hoxie JA, et al. Quantification of entry phenotypes of macrophage-tropic HIV-1 across a wide range of CD4 densities. J Virol. 2014; 88: 1858–1869. doi:10.1128/JVI.02477-13

75. Thomas ER, Dunfee RL, Stanton J, Bogdan D, Taylor J, Kunstman K, et al. Macrophage entry mediated by HIV Envs from brain and lymphoid tissues is determined by the capacity to use low CD4 levels and overall efficiency of fusion. Virology. 2007; 360: 105–119. doi:10.1016/j.virol.2006.09.036

76. Smith DH, Byrn RA, Marsters SA, Gregory T, Groopman JE, Capon DJ. Blocking of HIV-1 infectivity by a soluble, secreted form of the CD4 antigen. Science. 1987; 238: 1704–1707.

77. Li H, Wang S, Kong R, Ding W, Lee F-H, Parker Z, et al. Envelope residue 375 substitutions in simian-human immunodeficiency viruses enhance CD4 binding and replication in rhesus macaques. Proc Natl Acad Sci USA. 2016; 113: E3413–22. doi:10.1073/pnas.1606636113

78. Del Prete GQ, Keele BF, Fode J, Thummar K, Swanstrom AE, Rodriguez A, et al. A single gp120 residue can affect HIV-1 tropism in macaques. PLoS Pathog. 2017; 13: e1006572. doi:10.1371/journal.ppat.1006572

79. Kondo N, Miyauchi K, Meng F, Iwamoto A, Matsuda Z. Conformational changes of the HIV-1 envelope protein during membrane fusion are inhibited by the replacement of its membrane-spanning domain. J Biol Chem. 2010; 285: 14681–14688. doi:10.1074/jbc.M109.067090

80. Bibollet-Ruche F, Russell RM, Liu W, Stewart-Jones GBE, Sherrill-Mix S, Li Y, et al. CD4 receptor diversity in chimpanzees protects against SIV infection. Proc Natl Acad Sci USA. 2019; 11: 201821197. doi:10.1073/pnas.1821197116

81. Stephens M, Smith NJ, Donnelly P. A new statistical method for haplotype reconstruction from population data. Am J Hum Genet. 2001; 68: 978–989. doi:10.1086/319501

82. Stephens M, Donnelly P. A comparison of bayesian methods for haplotype reconstruction from population genotype data. Am J Hum Genet. 2003; 73: 1162–1169. doi:10.1086/379378

83. Librado P, Rozas J. DnaSP v5: a software for comprehensive analysis of DNA polymorphism data. Bioinformatics. 2009; 25: 1451–1452. doi:10.1093/bioinformatics/btp187

84. Malim MH, Bieniasz PD. HIV Restriction Factors and Mechanisms of Evasion. Cold Spring Harb Perspect Med. 2012; 2: a006940–a006940. doi:10.1101/cshperspect.a006940

85. Saito A, Akari H. Macaque-tropic human immunodeficiency virus type 1: breaking out of the host restriction factors. Front Microbiol. Frontiers; 2013; 4: 187. doi:10.3389/fmicb.2013.00187

86. Sawyer SL, Wu LI, Emerman M, Malik HS. Positive selection of primate TRIM5alpha identifies a critical species-specific retroviral restriction domain. Proc Natl Acad Sci USA. 2005; 102: 2832–2837. doi:10.1073/pnas.0409853102

87. Sawyer SL, Emerman M, Malik HS. Ancient adaptive evolution of the primate antiviral DNA-editing enzyme APOBEC3G. PLoS Biol. 2004; 2: E275. doi:10.1371/journal.pbio.0020275

88. McNatt MW, Zang T, Hatziioannou T, Bartlett M, Fofana IB, Johnson WE, et al. Species-Specific Activity of HIV-1 Vpu and Positive Selection of Tetherin Transmembrane Domain Variants. PLoS Pathog. 2009; 5: e1000300. doi:10.1371/journal.ppat.1000300

89. Lim ES, Malik HS, Emerman M. Ancient adaptive evolution of tetherin shaped the functions of Vpu and Nef in human immunodeficiency virus and primate lentiviruses. J Virol. 2010; 84: 7124–7134. doi:10.1128/JVI.00468-10

90. Longdon B, Brockhurst MA, Russell CA, Welch JJ, Jiggins FM. The evolution and genetics of virus host shifts. PLoS Pathog. 2014; 10: e1004395.

91. Woolhouse M, Scott F, Hudson Z, Howey R, Chase-Topping M. Human viruses: discovery and emergence. Phil Trans R Soc B. 2012; 367: 2864–2871.

92. Li W, Wong S-K, Li F, Kuhn JH, Huang I-C, Choe H, et al. Animal origins of the severe acute respiratory syndrome coronavirus: insight from ACE2-S-protein interactions. J Virol. 2006; 80: 4211–4219. doi:10.1128/JVI.80.9.4211-4219.2006

93. Fedeli C, Moreno H, Kunz S. Novel Insights into Cell Entry of Emerging Human Pathogenic Arenaviruses. J Mol Biol. 2018; 430: 1839–1852. doi:10.1016/j.jmb.2018.04.026

94. Kailasan S, Agbandje-McKenna M, Parrish CR. Parvovirus Family Conundrum: What Makes a Killer? Annu Rev Virol. 2015; 2: 425–450. doi:10.1146/annurev-virology-100114-055150

95. Arrildt KT, LaBranche CC, Joseph SB, Dukhovlinova EN, Graham WD, Ping L-H, et al. Phenotypic Correlates of HIV-1 Macrophage Tropism. J Virol. 2015; 89: 11294–11311. doi:10.1128/JVI.00946-15

96. Dunfee RL, Thomas ER, Gorry PR, Wang J, Taylor J, Kunstman K, et al. The HIV Env variant N283 enhances macrophage tropism and is associated with brain infection and dementia. Proc Natl Acad Sci USA. 2006;103: 15160–15165.

97. Laguette N, Sobhian B, Casartelli N, Ringeard M, Chable-Bessia C, Ségéral E, et al. SAMHD1 is the dendritic- and myeloid-cell-specific HIV-1 restriction factor counteracted by Vpx. Nature. 2011; 474: 654–657. doi:10.1038/nature10117

98. Berger G, Durand S, Fargier G, Nguyen X-N, Cordeil S, Bouaziz S, et al. APOBEC3A is a specific inhibitor of the early phases of HIV-1 infection in myeloid cells. PLoS Pathog. 2011; 7: e1002221.

99. O'Brien WA, Namazi A, Kalhor H, Mao SH, Zack JA, Chen IS. Kinetics of human immunodeficiency virus type 1 reverse transcription in blood mononuclear phagocytes are slowed by limitations of nucleotide precursors. J Virol. 1994; 68: 1258–1263.

100. Arfi V, Rivière L, Jarrosson-Wuillème L, Goujon C, Rigal D, Darlix J-L, et al. Characterization of the early steps of infection of primary blood monocytes by human immunodeficiency virus type 1. J Virol. 2008; 82: 6557–6565. doi:10.1128/JVI.02321-07

101. Kilareski EM, Shah S, Nonnemacher MR, Wigdahl B. Regulation of HIV-1 transcription in cells of the monocyte-macrophage lineage. Retrovirology. 2009; 6: 118. doi:10.1186/1742-4690-6-118

102. Joseph SB, Arrildt KT, Sturdevant CB, Swanstrom R. HIV-1 target cells in the CNS. J Neurovirol. 2015; 21: 276–289. doi:10.1007/s13365-014-0287-x

103. Schnell G, Joseph S, Spudich S, Price RW, Swanstrom R. HIV-1 replication in the central nervous system occurs in two distinct cell types. PLoS Pathog. 2011; 7: e1002286. doi:10.1371/journal.ppat.1002286

104. Cherner M, Masliah E, Ellis RJ, Marcotte TD, Moore DJ, Grant I, et al. Neurocognitive dysfunction predicts postmortem findings of HIV encephalitis. Neurology. 2002; 59: 1563–1567.

105. Antinori A, Arendt G, Becker JT, Brew BJ, Byrd DA, Cherner M, et al. Updated research nosology for HIV-associated neurocognitive disorders. Neurology. 2007; 69: 1789–1799. doi:10.1212/01.WNL.0000287431.88658.8b

106. Cai Y, Sugimoto C, Arainga M, Midkiff CC, Liu DX, Alvarez X, et al. Preferential Destruction of Interstitial Macrophages over Alveolar Macrophages as a Cause of Pulmonary Disease in Simian Immunodeficiency Virus-Infected Rhesus Macaques. J Immunol. 2015; 195: 4884–4891. doi:10.4049/jimmunol.1501194

107. Crowe SM, Westhorpe CLV, Mukhamedova N, Jaworowski A, Sviridov D, Bukrinsky M. The macrophage: the intersection between HIV infection and atherosclerosis. J Leukoc Biol. 2010; 87: 589–598. doi:10.1189/jlb.0809580

108. Stremlau M, Owens CM, Perron MJ, Kiessling M, Autissier P, Sodroski J. The cytoplasmic body component TRIM5alpha restricts HIV-1 infection in Old World monkeys. Nature. 2004; 427: 848–853. doi:10.1038/nature02343

109. Meyerson NR, Warren CJ, Vieira DASA, Diaz-Griferro F, Sawyer SL. Species-specific vulnerability of RanBP2 shaped the evolution of SIV as it transmitted in African apes. PLoS Pathog. 2018;14: e1006906. doi:10.1371/journal.ppat.1006906

110. Stabell AC, Hawkins J, Li M, Gao X, David M, Press WH, et al. Non-human Primate Schlafen11 Inhibits Production of Both Host and Viral Proteins. PLoS Pathog. 2016; 12: e1006066. doi:10.1371/journal.ppat.1006066

111. Nisole S, Lynch C, Stoye JP, Yap MW. A Trim5-cyclophilin A fusion protein found in owl monkey kidney cells can restrict HIV-1. Proc Natl Acad Sci USA. 2004; 101: 13324–13328. doi:10.1073/pnas.0404640101

112. Sayah DM, Sokolskaja E, Berthoux L, Luban J. Cyclophilin A retrotransposition into TRIM5 explains owl monkey resistance to HIV-1. Nature. 2004; 430: 569–573. doi:10.1038/nature02777

113. Cyranoski D. Monkey kingdom. Nature. 2016. 532 (7599): 300–302. doi:10.1038/532300a

114. Meyerson NR, Zhou L, Guo YR, Zhao C, Tao YJ, Krug RM, et al. Nuclear TRIM25 Specifically Targets Influenza Virus Ribonucleoproteins to Block the Onset of RNA Chain Elongation. Cell Host Microbe. 2017; 22: 627–638.e7. doi:10.1016/j.chom.2017.10.003

115. Lou DI, Kim ET, Meyerson NR, Pancholi NJ, Mohni KN, Enard D, et al. An Intrinsically Disordered Region of the DNA Repair Protein Nbs1 Is a Species-Specific Barrier to Herpes Simplex Virus 1 in Primates. Cell Host Microbe. 2016; 20: 178–188. doi:10.1016/j.chom.2016.07.003

116. Stabell AC, Meyerson NR, Gullberg RC, Gilchrist AR, Webb KJ, Old WM, et al. Dengue viruses cleave STING in humans but not in nonhuman primates, their presumed natural reservoir. eLife. 2018; 7: e01081–16. doi:10.7554/eLife.31919

117. Zhang J, Zhao J, Xu S, Li J, He S, Zeng Y, et al. Species-Specific Deamidation of cGAS by Herpes Simplex Virus UL37 Protein Facilitates Viral Replication. Cell Host Microbe. 2018; 24: 234–248.e5. doi:10.1016/j.chom.2018.07.004

118. Long EM, Rainwater SMJ, Lavreys L, Mandaliya K, Overbaugh J. HIV type 1 variants transmitted to women in Kenya require the CCR5 coreceptor for entry, regardless of the genetic complexity of the infecting virus. AIDS Res Hum Retroviruses. 2002; 18: 567–576. doi:10.1089/088922202753747914

119. Aricescu AR, Lu W, Jones EY. A time- and cost-efficient system for high-level protein production in mammalian cells. Acta Crystallogr D Biol Crystallogr. 2006; 62: 1243–1250. doi:10.1107/S0907444906029799

120. National Research Council (U.S.). Committee for the update of the Guide for the Care and Use of Laboratory Animals., Institute or Laboratory Animal Research (U.S.), National Academies Press (U.S.). 2011. Guide for the care and use of laboratory animals, 8^th^ ed.

121. Shang H, Han X, Shi X, Zuo T, Goldin M, Chen D, et al. Genetic and neutralization sensitivity of diverse HIV-1 env clones from chronically infected patients in China. J Biol Chem. 2011; 286: 14531–14541. doi:10.1074/jbc.M111.224527

122. Revilla A, Delgado E, Christian EC, Dalrymple J, Vega Y, Carrera C, et al. Construction and phenotypic characterization of HIV type 1 functional envelope clones of subtypes G and F. AIDS Res Hum Retroviruses. 2011;27: 889–901. doi:10.1089/AID.2010.0177

123. Rainwater SMJ, Wu X, Nduati R, Nedellec R, Mosier D, John-Stewart G, et al. Cloning and characterization of functional subtype A HIV-1 envelope variants transmitted through breastfeeding. Curr HIV Res. 2007;5: 189–197.
